# Comparative genomics, integrated with single-cell sequencing and genetic analyses, reveal roles of transcription factor AP2-M2 in asexual replication of *Babesia* parasite

**DOI:** 10.1371/journal.ppat.1013699

**Published:** 2025-11-13

**Authors:** Jinming Wang, Yijun Chai, Yuxin Ye, Jifei Yang, Xinxin Zhang, Hong Yin, Guiquan Guan

**Affiliations:** 1 State Key Laboratory for Animal Disease Control and Prevention, College of Veterinary Medicine, Lanzhou University, Lanzhou Veterinary Research Institute, Chinese Academy of Agricultural Sciences, Lanzhou, Gansu, China; 2 Key Laboratory of Veterinary Parasitology of Gansu Province, Gansu Province Research Center for Basic Disciplines of Pathogen Biology, Lanzhou, Gansu, China; 3 Jiangsu Co-Innovation Center for the Prevention and Control of Important Animal Infectious Disease and Zoonoses, Yangzhou University, Yangzhou, China; Pennsylvania State University Main Campus: The Pennsylvania State University - University Park Campus, UNITED STATES OF AMERICA

## Abstract

Babesiosis is an emerging and re-emerging zoonosis that is prevalent worldwide, caused by over 100 *Babesia* species. These intracellular parasites efficiently invade host red bloods cells, replicate rapidly, and exit the infected cells to cause clinical symptoms. However, the underlying mechanisms of regulating this asexual stage is largely unknown. Here, we generated a chromosome-level reference assembly of a novel *Babesia* species, *Babesia xinjiangensis*. Using single-copy orthologous genes, we confirmed its phylogenetic relationships with other apicomplexan parasites and estimated its speciation time. We identified species-specific gene families and core gene families that could be responsible for species speciation and immune evasion. Furthermore, we also used a single-cell RNA-sequencing (scRNA-seq) protocol to uncover hidden transcriptional variations in the asexual stages of this unicellular *Babesia* parasite and its cell-to-cell heterogeneity. We inferred the replication cycle and performed a pseudotime analysis to speculate the gene expression profiles. Although the peak expression times of most epigenetic markers and transcription factors were confined to specific phases, BxAP2-M2 (GWHPERCV002055) is constantly expressed during asexual development progression. Genetic analyses revealed that BxAP2-M2 directly or indirectly regulates the expression of rhoptry proteins and membrane proteins, which may play critical roles in the parasite’s invasion of red blood cells and the merozoite morphology. Our findings provide valuable markers of asexual replication, including some that are specific to *Babesia* gametocytes, and regulators specific to distinct cell-cycle phases.

## Author summary

Understanding the molecular mechanisms regulating the asexual replication of *Babesia* parasite is critical for developing effective strategies to control babesiosis, and therefore has significant veterinary and public health implications. In this study, we combined comparative genomics, single-cell RNA sequencing, and genetic analyses to determine the regulatory networks governing the asexual replicative cycle of *Babesia xinjiangensis*, a novel ovine parasite. We identified key transcription factors, including BxAP2-M2, that regulate red blood cell invasion and parasite development. Investigating the gene expression dynamics during different developmental stages provided novel insights into the transcriptional regulation of *Babesia* replication. These findings not only enhance our understanding of *Babesia* biology but also offer potential targets for therapeutic interventions aimed at disrupting the parasite’s replication cycle, which will ultimately contribute to the control of this economically and medically important pathogen.

## Introduction

Babesiosis is a substantial emerging and re-emerging disease caused by parasites of the genus of *Babesia* in the phylum Apicomplexa, which are mainly transmitted via the bites of their tick vectors or less commonly by blood product transfusion or solid organ transplantation [1]. The significant veterinary and agricultural importance of this disease has long been recognized, because it causes hundreds of millions of dollars of economic losses each year [2,3]. Up to now, more than 100 *Babesia* species have been documented, which infect almost all vertebrate hosts, including wild animals, domestic animals, and humans. The clinical symptoms of human babesiosis range from fever, headache, and multi-system organ failure to death [3,4]. With the expansion of human activities and the changes in environmental factors, increasing *Babesia* species are reported to cause human babesiosis, including *Babesia microti*, *Babesia divergens*, *Babesia duncani*, *Babesia crassa*, and *Babesia motasi* [5–10]. Ovine babesiosis, predominantly caused by *Babesia ovis*, two *Babesia motasi* subspecies, *B. crassa*, and *Babesia* sp. Xinjiang, often leads to significant economic agricultural losses and poses a risk to human health [11–13].

The complex life cycle of *Babesia* species involves a strictly regulated progression: intraerythrocytic asexual replication in the vertebrate host and sexual differentiation and development in tick vectors [14–16]. When an infected tick attaches to vertebrate host, the dormant sporoblast is activated, and infective sporozoites bud off from the syncytium and are released in blood during tick feeding [16]. Then they invade red blood cells and trigger asexual merogony, then the progenies of merozoites egress from the infected red blood cells, and re-invade adjacent erythrocytes to increase the size of the population [14]. One round of this intraerythrocytic life cycle is completed every 6.4-26.4 hours, varying across different species [17–21]. During the asexual replication stages, a small proportion of merozoites differentiates into gametocytes, which develop in the tick vectors and produce sporozoites that can propagate in a new vertebrate host [16].

Morphological changes during the asexual replicative cycle of *Babesia* species are accompanied by varying transcriptional profiles. The bulk transcriptomes of various *Babesia* species have been analyzed [22,23]. However, only limited studies have analyzed synchronized parasites from in vitro cultures, and the transcriptional profiles during the asexual replication of most species are not currently available [24]. Furthermore, the data from samples collected at different time points during the development of strictly synchronized cultures do not reflect the real changes in transcriptional profiles and not always accurately capture gene expression patterns. Our understanding of the molecular mechanisms underlying the asexual replication of *Babesia* species has mainly been gained from the comparative analysis of other apicomplexan parasites, including *Plasmodium* spp. and *Toxoplasma gondii* [25–27]. This information is inadequate in light of the deep evolutionary divergence of these species and the significant differences in their biology, including their distinct microscopic morphologies, pathogenesis, host specificity, and transmission vectors.

Advances in genomic sequencing technologies and the substantial development of analytical platforms allow us to determine the gene expression profiles of individual cells, and provide powerful tools for dissecting the heterogeneity of unicellular organisms and gain insights into precise regulation of gene expression in *Babesia* parasites [28]. The long read sequencing technology has made the chromosome-level genome of *B. duncani* available, to better understand its biological features [6,28]. Single-cell transcriptional sequencing (scRNA-seq) technology has been successfully employed to illustrate whole cell populations in the entire life cycle of *Plasmodium* spp., the asexual replication stages of three *Babesia* species, as well as the mechanisms regulating their asexual and sexual developments. *Babesia* sp. Xinjiang was firstly isolated from a splenectomied sheep infested with partially engorged *Rhipicephalus sanguineus* and *Hyalomma anatolicum* in Xinjiang Uygur Autonomous Region in China [12,17,29–31]. Systematic analyses of this *Babesia* species, including its morphological characteristics, transmission patterns, culture features, epidemiology, pathogenicity, and whole genomic annotation have confirmed it as a novel species, distinct from other *Babesia* species that infect sheep. It was designated ‘*Babesia xinjiangensis*’. Here, we provide the chromosome-level genome of this species and characterized transcriptional profiles and potential regulators of its asexual replication. Genetic analyses showed that an AP2 transcription factor, BxAP2-M2, plays significant roles in regulating the expression of rhoptry proteins and modulating the efficiency of host cell invasion.

## Results

### *Babesia xinjiangensis* genome assembly

**Fig 1:**
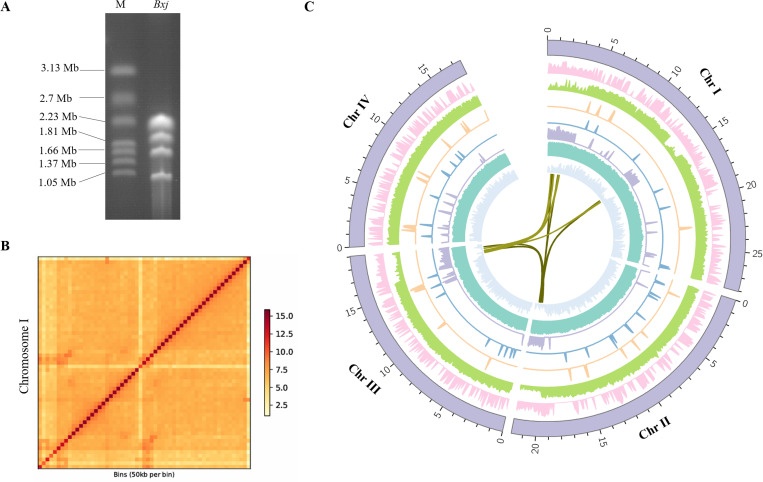
Genomic features of *Babesia xinjiangensis.* **(A)** Plused-field gene electrophoresis (PFGE) showing the number and approximate sizes of chromosomes (Chrs); M, *Hansenula wingei* genome; Bxj, *Babesia xinjiangensis* genome. **(B)** Hi-C chromosomal contact maps of *B. xinjiangensis* chromosome (Chr) **I. (C)** Circular plot of the genome organization of *B. xinjiangensis*. From the innermost to outermost ring: gene synteny, density of gene model, % of GC content, *vesa* expression levels, density of short interspersed nuclear elements, locations and expression levels of epigenetic markers, gene expression levels, levels of novel gene expression, scaffold size. Chrs are divided into 100-kb bins.

To produce an improved reference genome, *B. xinjiangensis* merozoites were purified from a culture of the parasite in ovine erythrocytes. A chromosome-level genome assembly was generated from long reads acquired with the Oxford Nanopore Technology (ONT) long reads (~200-fold coverage). The assembly was polished with ONT long reads and short paired reads on the Illumina sequencing platform, which were used to correct the short insertions, deletions, and substitutions in the assembly generated from the ONT reads. A pulsed-field gel electrophoresis (PFEG) analysis revealed that the *B. xinjiangensis* genome has four chromosomes (Fig 1A). Our assembly contained eight scaffolds, three of which had acrocentric centromere (A similar genome structure has also observed in *B. bovis* and *B. duncani*). To further anchor the contigs of the assembly to the chromosomes, they were ordered and oriented to the chromosomes with the Hi-C data with the ALLHiC software package (Fig 1B). The genome size of *B. xinjiangensis* was 8.6 Mb, which included four chromosomes of 2.8 Mb (Chromosome (Chr) I), 2.2 Mb (Chr II), 1.9 Mb (Chr III), 1.7 Mb (Chr IV) (Fig 1B and 1C). The completeness of the genome, evaluated with BUSCO (v5.1.3) using the core apicomplexan dataset (apicomplexa_odb10), was 95.5% (94.8% single-copy genes and 0.7% duplicated genes) (S1A Fig) [32]. The size of the *B. xinjiangensis* genome, like those of *B. microti*, *B. duncani*, and *B. bovis*, is smaller than those of *B. motasi lintanensis*, *B. motasi hebeiensis*, *B. ovata*, and *B. divergens* (basic information on the *B. xinjiangensis* genome is provided in Table 1).

**Table 1:** Basic information of *Babesia* spp. genomes.

	*B. xinjiangensis*	*B. motasi hebeiensis*	*B. motasi lintanensis*	*B. bovis*	*B. duncani*	*B. microti*
Genome size (Mb)	8.7 Mb	14.6	14.6	8.2	7.9	6.4
N50	1,766,331 bp	3045872	2883146	1797577	1067452	1766409
GC (%)	43.52	46.96	47.28	41.69	37.68	36.17
Coding gene numbers	3,771	4150	4432	3974	3771	3573
N’s per 100 kbp	0.46	0	0.03	0.01	0.01	1.59
Gene density (gene/Mb)	435.5	284.2	303.6	484.6	477.3	558.3
Number of exons per gene	2.78	2.7	2.7	2.8	2.7	7.7
Genes contained multi-exon	2,314					
Completeness of predicted genes (BUSCO)	96.80%					
Completeness of the genome (BUSCO)	95.50%	97.50%	96.80%	96.90%	95.30%	94.80%

### Annotation of *B. xinjiangensis*

To more reliably predict the gene models, Illumina RNA-seq reads were generated from RNA isolated from merozoites cultured in vitro in ovine erythrocytes. We also used *de novo* prediction with both Augustus and GlimmerHMM and a homology-alignment with apicomplexan parasite genes to predict the gene models [33–35]. We predicted 3,771 protein-coding genes with an average gene length of 1,458 bp, 61.2% (2,314/3,771) of which contained multiple exons. The functional annotation of 81.6% of these genes was performed with BLASTP and Interproscan. The completeness of the predicted gene set was determined to be 96.8% (96.4% single-copy genes and 0.4% duplicated genes; S1A Fig) by mapping it against the genes in the apicomplexa_odb10 database. Compared with the previous version (GCA_002095265.1), ~ 700 novel genes (average length of 2,658 bp) were identified (Fig 1C). The *B. xinjiangensis* genome encodes 46 tRNAs almost evenly distributed between four chromosomes with 10 on Chr I, 11 on Chr II, 11 Chr III, and 14 on Chr IV.

### Phylogenetic relationships and comparison of *B. xinjiangensis* with other apicomplexan parasites

**Fig 2:**
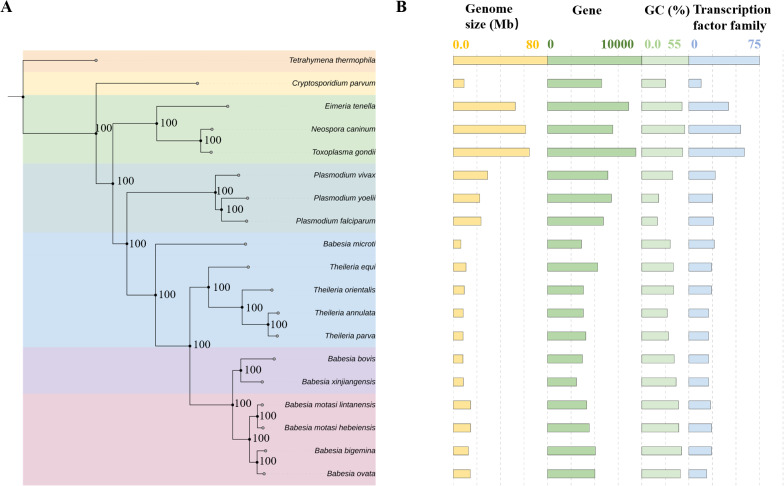
Phylogenetic analysis of *Babesia xinjiangensis* and other Apicomplexan parasites. **(A)** Phylogenetic relationships inferred for 18 species of Apicomplexa parasites and an outgroup, after the alignment of 2,929,403 amino acids from 321 single-copy orthologues. **(B)** Genome sizes, numbers of predicted protein-coding genes, GC contents, and transcription factor gene families across Apicomplexan parasites are shown in bar plots.

To accurately determine the evolutionary distances and relationships between *B. xinjiangensis* and other apicomplexan parasites, a phylogenetic analysis of 321 one-to-one single-copy orthologous genes from 19 species across the Apicomplexa was used to construct a species tree (S1 Table). *Tetrahymena thermophila* was used as the outgroup. The results of this analysis were consistent with those of a previous study of piroplasm classification based on single-copy orthologous genes. *Babesia xinjiangensis*, together with *B. bovis*, clustered to clade II, which shared a common ancestor and sister-group relationships with clade Ⅲ (*B. m. hebeiensis* and *B. m. lintanensis*) and clade IV (*B. bigemina* and *B. ovata*) (Fig 2A).

The divergence times of apicomplexan parasites were calculated based on amino acid mutation rate using MCMCTree [36,37], calibrated with the following divergence times: the divergence of *Toxoplasma gondii* and *Plasmodium falciparum* 817 million years ago (Mya), 95% highest posterior density intervals (HPD) of 580–817 Mya), the divergence of *Eimeria tenella* and *Neospora caninum* at 470 Mya, and the divergence of *Cryptosporidium parvum* and *Te. thermophila* at 1,290 Mya, 95% HPD of 767–344 Mya. The *Babesia* spp. belonging to clade Ⅲ and clade IV shared an ancestor with *B. xinjiangensis* around 233.3 Mya, and *B. xinjiangensis* was more closed related to *B. bovis* than to other piroplasm parasites. The ancestor of *B. xinjiangensis* and *B. bovis* split into two distinct species around 165.1 Mya, with capabilities to infect ovine and bovine hosts, respectively (S1B Fig).

The total of 3,771 *B. xinjiangensis* genes are evenly distributed across the four chromosomes, with 1,145 on Chr I, 960 on Chr II, 870 Chr III, and 796 on Chr IV. The results of GC content, gene density and sequence coverage of *B. xinjiangensis* are shown in Fig 1c. the basic features of *B. xinjiangensis* genome and gene number of transcription factor family are provided in Fig 2b. Using the OrthoFinder platform to detect orthologous genes, we identified 949 core genes that are commonly present in Apicomplexa, whose possible functions have been described in other species. *Babesia xinjiangensis* also contains 279 unique genes (identified only in *B. xinjiangensis*), 33.9% of which were predicted to encode uncharacterized proteins, suggesting that their precise functions warrant investigation. *Babesia xinjiangensis* shares 3,641 homologous with of *B. bovis*, but fewer with *B. m. hebeiensis* (*n = *3,338). A synteny analysis confirmed that *B. xinjiangensis* is more similar to *B. bovis* than to *B. m. hebeiensis* (S1C Fig).

### Multigene families of *B. xinjiangensis*

We identified 495 genes belonging to multigene families in the *B. xinjiangensis* genome, with each family containing at least three members. We assigned these families to two groups: those existing in at least two other apicomplexan parasites (BxjE) and those unique to *B. xinjiangensis* (BxjU). BxjU contained 242 genes, which were divided into 14 gene families, whereas BxjE contained 253 genes, which were classified into 54 gene families, each corresponding to an orthologue in the apicomplexans (S1 Table). The chromosomal distributions of each gene family and their members are shown in Fig 1 and S1 Table, respectively. The chromosomal locations of the families in BxjE (variant erythrocyte surface antigen [VESA] family, three transcription factor [TF] gene families, including myeloblastosis [MYB], high mobility group [HMG], and Apetala2 [AP2], and histone methyltransferase [HMT]) are shown in Fig 1c. The *B. xinjiangensis* genome contains 150 VESA-encoding genes, 92 of which are predominately enriched in the subtelomeres or centromeres of Chr I, and only a small number are located in subtelomeres of Chr II (*n = *26) or Chr III (*n = *21). 11 VESA-encoding genes are dispersed on Chr IV. The TF and HMT gene families are uniformly distributed across the four chromosomes (S1 Table).

Among the 14 gene families of BxjU, the distribution of the BxjUF1, BxjUF2, and BxjUF3 families, located near the telomeres, subtelomeres, or centromeres, is similar to that of the *var* genes of *P. falciparum*, the *Blf1* of *B. duncani*, and VESA of B. bovis, respectively, suggesting that they may associate with parasite-host interaction and immune evasion [6,38,39]. These three gene families (BxjUF1, BxjUF2, and BxjUF3)) contain 95, 49, and 37 members, respectively, with no significant sequence similarity to genes of other apicomplexan parasites (S1 Table and S2A Fig). Many members of BxjUF1 (49/95) and BxjUF2 (23/49) contain multiple exons. However, only one member of BxjUF3 consists of multiple exons. When the members of each family were comparted, low levels of sequence similarity were observed within each family. However, members of each family shared conserved amino acid motifs and some conserved regions (S2B Fig).

### Gene expression profiles of *B. xinjiangensis* during asexual development

**Fig 3:**
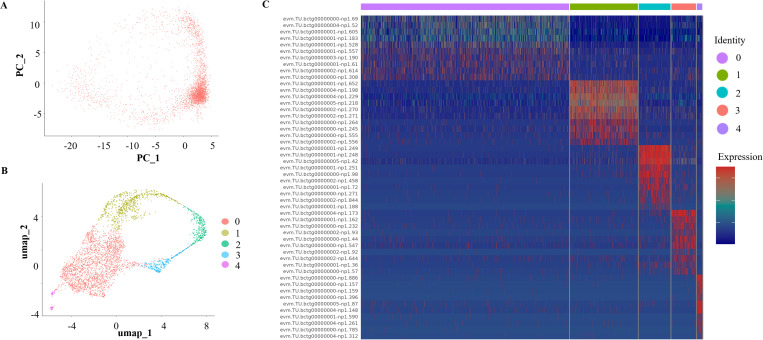
A single cell atlas of the asexual reproduction of *Babesia xinjiangensis.* **(A)** Principal components analysis (PCA) of *B. xinjiangensis* transcriptome; each dot represents a single parasite transcriptome. **(B)** Uniform manifold approximation and projections (UMAP) of single-cell transcriptomes sampled from asexual stages, with cells colored according to their transcriptomic patterns. **(C)** Heat map of the mean expression of the top 10 differentially expressed markers in each cluster.

**Fig 4:**
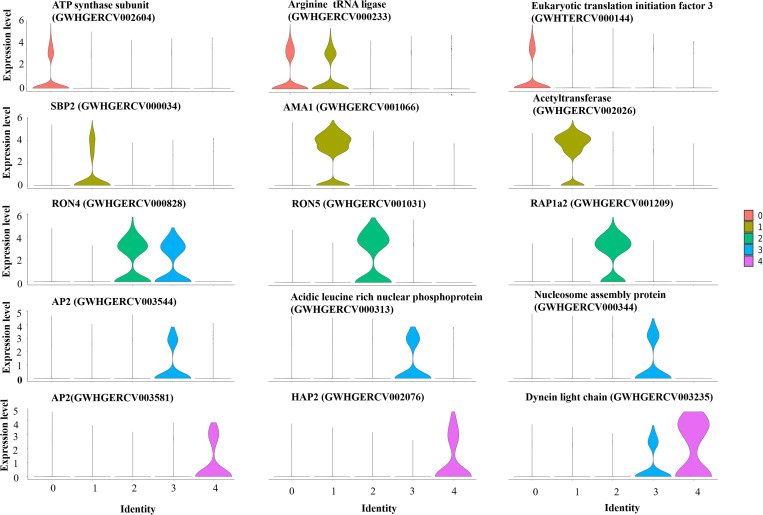
Comparative analysis of marker genes for each cluster. Violin plot shows the levels of average gene expression corresponding to each cluster.

To investigate the gene expression profiles of *B. xinjiangensis* during asexual reproduction, we prepared two scRNA-seq library using cultures of the parasite in sheep erythrocytes. Transcripts abundance values were produced by processing the scRNA-seq data with the Seurat package (v5.0). The alignment rates of the scRNA-seq data with the *B. xinjiangensis* genome were > 85%. We observed that approximately 15% of reads were mapped to the ovine genome, which is consistent with the low abundance of nucleic acid content typically found in red blood cells. A total of 3,933 cells and 3,442 genes were detected in *B. xinjiangensis*; and 408 (median genes per cell) genes exceeded the Seurat cutoff. A principal component analysis (PCA) of the levels of gene expression showed that *B. xinjiangensis* consisted of distinct populations; 3,933 cells were grouped into five classes using uniform manifold approximation and projection (UMAP), and showed a circular pattern of expression, similar to those in *B. bovis*, *B. bigemina*, and *B. divergens* (Fig 3A and 3B) [28]. Each cluster was compared against the remaining four to identify significantly differentially expressed genes. We identified 200 significantly differentially expressed genes (DEG; fold change > 1.5 and adjusted *p*-value < 0.01), which were associated with metabolism, chromosome replication, and organelle biogenesis (Fig 3c and S2 Table). The DEGs of *Babesia* spp. (*B. bovis*, *B. bigemina*, and *B. divergens*) in each phase of the cell cycle were used to identify the cluster features of *B. xinjiangensis* [28]. Cluster 0 in *B. xinjiangensis* was enriched for protein synthesis and energy metabolism, including genes related to glycerol 3-phosphate dehydrogenase (GWHGERCV000210), tRNA ligase (GWHGERCV001598, GWHGERCV002787), protein kinase (GWHGERCV001390, GWHGERCV003018, GWHGERCV003171, GWHGERCV003046, and GWHGERCV000493), eukaryotic translation initiation factor (GWHGERCV003551), and ATP synthase (GWHGERCV003597). The cluster 1 genes were associated with micronemes and spherical-body secretory organelles, including thrombospondin related adhesive proteins (TRAPs, GWHGERCV000243 and GWHGERCV000245), apical membrane antigen 1 (AMA1, GWHGERCV001066), gliding associated protein 45 (GAP45, GWHGERCV000497), and spherical body proteins (SBPs, GWHGERCV000034, GWHGERCV001560 and GWHGERCV003270). *Plasmodium falciparum* merozoite-specific TRAP (MTRAP) and AMA1 interact with host receptors Semaphorin-7A and Kx, respectively, likely to modulate the invasion process. Meanwhile, the parasite protein RON2 serves as the primary receptor for AMA1, forming the moving junction [40,41]. SBPs involved in parasite virulence and invasion process of merozoite [42,43]. The genes in cluster 2 were enriched for those encoding rhoptry proteins: rhoptry neck protein 4 (RON4, GWHGERCV000828), RON 5 (GWHGERCV001031), RON 11, rhoptry associated protein 1 (RAP1, GWHGERCV001206), RAP1α (GWHGERCV001207), RAP1α2 (GWHGERCV001209), RAP-1 related antigen (RRA, GWHGERCV001164), Rhoptry proteins secreted by the parasite play critical roles in host cell invasion and survival. Among them, RON4 and RON 5 are key components of moving junction, a structure formed between the apical end of the merozoite and the red blood cell surface that facilitates invasion [44]. This cluster was also enriched for the egress-related proteins, aspartyl protease 3 (ASP3, GWHGERCV001398), rhomboid 4 (GWHGERCV000548), cyclin associated kinase (GWHGERCV003283), and calcium dependent protein kinase 4 (GWHGERCV001988). The genes of cluster 3 were associated with the inner membrane complex, membrane skeletal, and nucleosome components, including membrane skeletal protein (GWHGERCV002472), integral membrane protein (GWHGERCV000925 and GWHGERCV003711), histone 2A/2B/3 (GWHGERCV000135, GWHGERCV000150, and GWHGERCV000151), and nucleosome assembly protein (GWHGERCV000344). Interestingly, the cluster 4 contained strongly expressed genes encoding orthologues of gametocyte protein (HAP2, GWHGERCV002076) and dynein light chain (GWHGERCV003235), which are uniquely expressed on *B. bovis* sexual developmental stages in ticks and expressed in mature male gametocytes of *Plasmodium falciparum*, respectively [45–47]. Therefore, this cluster was designed as gametocyte, and has not been observed in other *Babesia* species during intraerythrocytic asexual development (Fig 4 and S2 Table). These clusters showed distinct transcriptional patterns in parasitic cells proceeding from the post invasion stage, to the fast growth of merozoites, and to the to pre-egress stage.

**Fig 5:**
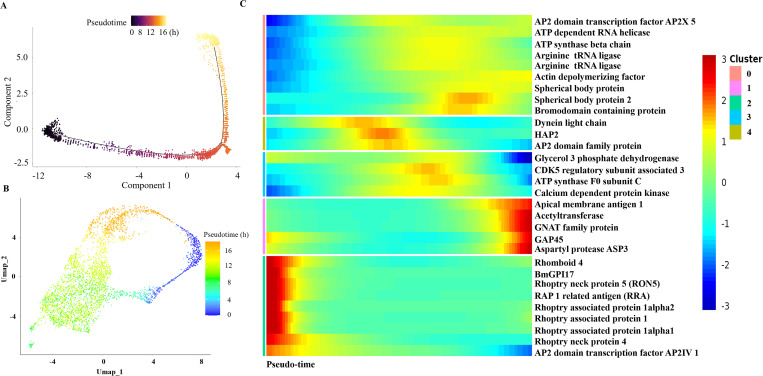
Pseudotime and dynamic gene expression over pseudo-time. **(A)** Dimensionality reduction of pseudotime. **(B)** Uniform manifold approximation and projections (UMAP) representation of potential developmental trajectories of *Babesia xinjiangensis*. **(C)** Heatmap of the dynamic gene expression over trajectories of the parasite’s asexual development.

To further investigate the differential gene expression during asexual replication, we carried out discriminative dimensionality reduction in a pseudotime analysis. We defined 0 h as the start timepoint and 16 h as the terminal timepoint, representing the approximate duration of a complete division cycle in *B. xinjiangensis* [17]. The developmental trajectories were inferred from the pseudotime analysis by comparing the transcriptome of each cell and placing those cells with similar gene expression profiles closer to each other. We compared transcriptome of each cell and performed the pseudotime analysis with Monocle 2, which has been used successfully to speculate the developmental trajectories of other apicomplexan parasites (e.g., *Plasmodium berghei*, *P. falciparum*, and *Babesia* spp.), suggesting that apicomplexan parasites can be accurately ordered by their transcriptional features [25,26,28]. The order estimated with the pseudotime analysis was consistent with previously reported bulk RNA-seq data collected during a time-course of development [25,26,28]. The reconstructed developmental trajectories towards mature merozoites and gametocytes were composed of two terminal branches, reflecting three main cell states (Fig 5A and 5B).

In obvious contrast to the abrupt transitions observed in the cell-cycle processes of *P. berghei* and *P. falciparum*, we observed smooth changes in gene transcription during the asexual reproduction of *B. xinjiangensis* [25,48]. Gene expression smooth changes support the continuous development in the intraerythrocytic life cycle of this parasite, supported by the continuous distribution of parasites in the PCA plot. We also examined previously published data for *Babesia* spp. and found profiles similar to those of *B. xinjiangensis*. the step-wise progression of the cycle showed a conserved pattern of transcription across Apicomplexa. Individual cells did not show a continuous increase in transcript abundance, but rather shifts in transcription, turning on or shutting down specific gene repertoires (Fig 5C). These patterns of transcription were turned on or turned off smoothly during reproduction, although the two patterns could coexist in the same cluster.

To illustrate the biological variance that characterized the cell states, we performed UMAP dimensionality reduction to map cell states and visualize cellular distributions in low-dimensional space. The variance in the transcriptional patterns in defined cell states reflected the differential transcriptional features of merozoites and gametocyte, which differ from their common progenitor cells. To capture the differential RNA profiles that defined the heterogeneity of the cell states, we employed the Wilcoxon test to analyze the differential gene expression. In our putative trajectory (computationally inferred path representing the dynamic transitions of cell states based on transcriptional similarities between cells), we observed that states 1 (clusters 1 and 2) and 2 (cluster 3) featured in distinct transcriptional signatures during merozoite development, including the merozoite markers related to host cell invasion (e.g., rhoptry-associated proteins, spherical body proteins) and genes involved in egress (e.g., aspartyl protease and protein kinase). State 3 (cluster 4) was characterized by previously identified markers of *B. bovis* and *P. falciparum* gametocytes, encoding HAP and dynein heavy chain, which were strongly upregulated genes (Fig 5B and S2 Table). This suggested that a small population of gametocytes was generated during the asexual stages. Stage 3 also included genes encoding the transcription factors GWHGERCV002182 and GWHGERCV003581 (AP2X-5), which may play potential roles in regulating gametocyte differentiation.

**Fig 6:**
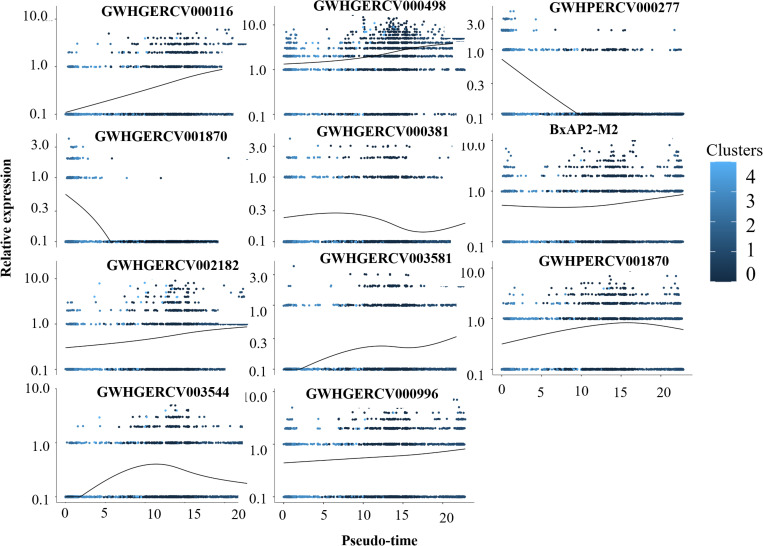
Scaled expression levels of specific genes as a function of pseudotime.

Temporal gene expression throughout the lifecycle is regulated by transcription factors that have been validated to been conserved across Apicomplexa [49–51]. To assess the transcriptional activity of the Apetala2/ethylene response factor (AP2) family during asexual replication, we analyzed the transcriptional dynamics of the AP2 genes and inferred the trends in their expression in various cell states. An undefined functional AP2 gene was observed to be expressed in gametocytes. We observed expression changes in the expression of several AP2 genes, including GWHGERCV000116, GWHGERCV000381, GWHGERCV000498, GWHGERCV000996, GWHGERCV001870, GWHGERCV002182, GWHGERCV003544 (AP2 IV-1), and GWHGERCV003581, that were associated with the development of merozoites or with gametocytogensis (Fig 6). These findings indicate that the expression of a given AP2 gene may correlate with a specific stage of reproductive progression. As well as the enrichment of those genes in specific clusters, we noted that a transcription factor (BxAP2-M2, GWHPERCV002055) was expressed almost consistently throughout merozoite development, implicating it in modulation of these gene expression patterns.

### Transcription factor BxAP2-M2 regulates merozoite morphology and invasion processes

**Fig 7:**
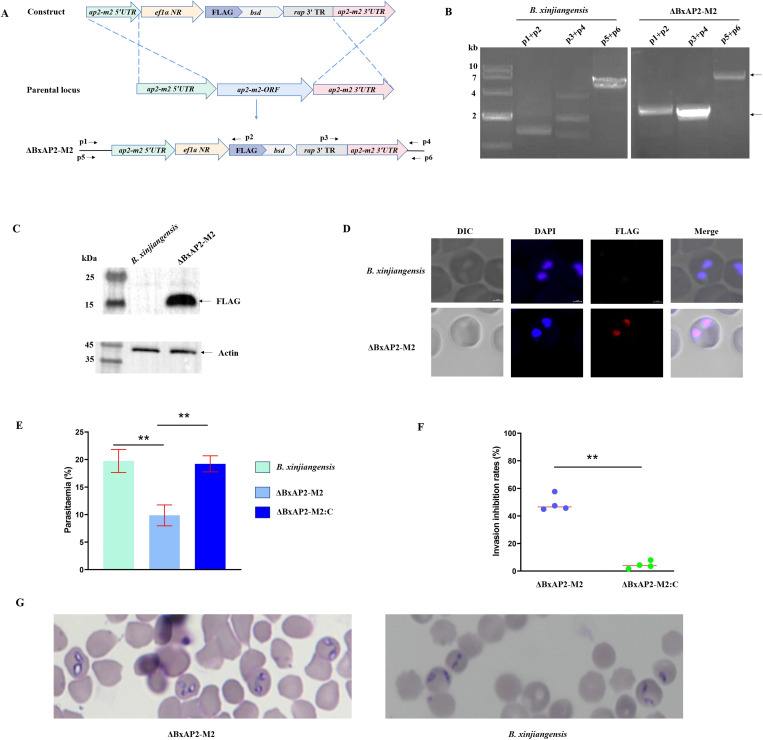
Functional analysis of BxAP2-M2 during the asexual development of *Babesia xinjiangensis.* **(A)** Diagram illustrating the construction a BxAP2-M2-disrupted strain (ΔBxAP2-M2). **(B-D)** ΔBxAP2-M2 strain confirmed with PCR, western blotting, and immunofluorescence assay, respectively. The amplified fragments generated by primer pairs p1/p2, p3/p4, and p5/p6 were 2,098 bp, 1,998 bp, and 6,421 bp in length, respectively. The expected molecular weight of the FLAG-BSD fusion protein is 14.5 kDa. **(E)** Significantly reduced growth rate observed in ΔBxAP2-M2. *P* value was generated using a one-way ANOVA test. **P < 0.05. The error bars represented standard deviation from three independent experiments. **(F)**, Reduced invasion efficiency in the ΔBxAP2-M2 parasite. *P* value was generated using a one-tailed Student’s t-test. **P < 0.05. **(G)** Giemsa-stained images showing morphological changes in 47% of ΔBxAP2-M2 merozoite. ef1α NR, ef1α promotor region; FLAG, FLAG tag; bsd, Blasticidin S resistance gene; rap 3’ TR, terminal region of rhoptry associated protein gene; ap2-m2 5’ UTR, promotor region of ap2-m2 gene; ap2-m2 3’ UTR, terminal region of ap2-m2 gene.

The BxAP2-M2 gene encodes a protein containing 355 amino acids, with one AP2 domain. Although homologues of BxAP2-M2 protein have been identified in several apicomplexan parasites, including *B. microti*, *B. bovis*, *B. bigemina*, and *Theileria* spp., their roles in these species have not been described (S3 Fig). To investigate the biological function of *B. xinjiangensis* BxAP2-M2, we directly knocked it out using homologous gene replacement with the selective marker blasticidin S deaminase (BSD), which generates a parasite strain resistance to this drug (Fig 7A-7D). A single clone of *B. xinjiangensis* was obtained with limiting dilution, indicating that BxAP2-M2 is not essential for the survival of the parasite. To further characterize the associated phenotype, we also generated a genetic complementation strain by expressing an HA-tagged BxAP2-M2 in the *BxAP2-M2* knockout background, driven by the ef1α promoter at the endogenous *ef1*α*B* locus (S4 Fig). We evaluated the role of BxAP2-M2 during the process of parasite invasion and found that the invasion efficiency of ΔBxAP2-M2 was significantly reduced (by 48.9 ± 5.9%) relative to that of the strain expressing BxAP2-M2 (Fig 7E and 7F). In 47% of merozoites, the morphology of ΔBxAP2-M2 changed from “slim paired piriform” to “fat paired piriform” (Fig 7G). These abnormalities were almost completely reversed in the ΔBxAP2-M2-complemented strain. After 12 hours of merozoites invasion, We observed that 82.4 ± 3.9% and 80.3 ± 4.4% of merozoites developed into paired piriform in wild type strain and ΔBxAP2-M2, respectively. Approximately 15% of single piriform is observed in vitro culture system of *B. xinjaingensis* (~15%). These results indicated that disruption of BxAP2-M2 has no effect on merozoite development (*t* = 1.09, *df* = 2, *P*-value = 0.39). Furthermore, we evaluated the egress efficiency and found no significant difference (*t* = 0.42, *df* = 4, *P*-value = 0.70) between the *B. xinjiangensis* (egress efficiency: 31.32 ± 6.38%) and ΔBxAP2-M2 (egress efficiency: 29.03 ± 7.00%) (S3 Table). Taken together, these results indicate that BxAP2-M2 is important for *B. xinjiangensis* growth, but not essential for its survival.

### Transcription factor BxAP2-M2 regulates gene expression involved in morphology and invasion processes

**Fig 8:**
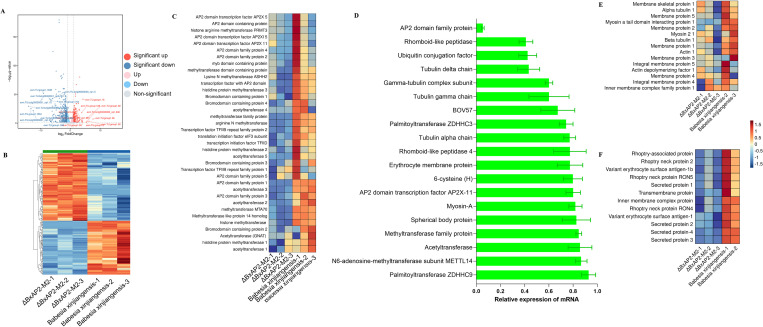
Gene expression profiles of BxAP2-M2-disrupted strain. **(A)** Volcano plot of the differentially expressed genes between ΔBxAP2-M2 and *Babesia xinjiangensis.* Data from three biological replicates were analyzed; fold change > 2.0 and *p*-value < 0.05 were used as cutoffs to identify differentially expressed genes. **(B)** Heat map of differentially expressed proteins. A fold change > 2.0 and a *p*-value <0.05 were used as cutoffs to identify differentially expressed proteins. **(C)** Differentially expressed genes involved in epigenetic regulation. **(D)** Differentially expressed genes were validated with real-time PCR. The error bars represented standard deviation from three independent experiments. **(E)** Proteins involved in maintaining merozoite morphology. **(F)** Proteins associated with merozoite invasion.

To determine the molecular mechanisms underlying the poor invasion efficiency of and morphological changes in ΔBxAP2-M2, we compared the transcriptional patterns of ΔBxAP2-M2 with those of *B. xinjiangensis*. An RNA-seq analysis revealed that the transcription of 1,435 genes was reduced in ΔBxAP2-M2, and that the transcription of 265 of these genes was significantly decreased (fold change > 2.0; *P*-value < 0.05) (Fig 8A and 8B and S4 Table). Among the significantly downregulated genes, 37.7% (100 out of 265) were predicted to be unrelated to merozoite morphology and invasion efficiency. These include conserved *Plasmodium* proteins, uncharacterized proteins, and ribosomal subunit proteins. Significantly difference genes were performed Gene Ontology (GO) enrichment and Kyoto Encyclopedia of Genes and Genomes (KEGG) pathway analyses. The dysregulated genes showed significant enrichment in terms related to biological processes, cellular components, and molecular functions (S5A Fig). However, these enriched pathways did not correlate with our observed decrease in host cell invasion efficiency and the morphological change. These genes implicated in host cell invasion—such as those encoding rhoptry neck proteins, rhoptry-associated proteins, spherical body proteins, bov57, and merozoite surface proteins—could not be effectively annotated in GO and KEGG databases (S5B Fig). We observed that 1,469 genes exhibited elevated expression levels, among which 198 genes showed statistically significant transcriptional upregulation. We noted that the expression of genes encoding secreted-organelle coding genes, including spherical body protein, bov57, and merozoite surface proteins, was downregulated. The changes in the transcription of these genes were confirmed with real-time PCR (RT-PCR), which showed a > 2.0-fold reduction in ΔBxAP2-M2 (Fig 8C and 8D and S5 Table). Among the 89 significantly down-regulated proteins, only a small proportion (5/89) have been validated as involved in the invasion process, with an additional 11 proteins predicted to be secreted or uncharacterized (S6 Table). Importantly, the expression levels of the rhoptry proteins (RON2, RON4, RON5, RAP1) in strain ΔBxAP2-M2 were significantly decreased, when determined with label-free ultra-high-pressure liquid chromatography and mass spectrometry. Rhoptry proteins are critical components of moving junctions, which play an essential role during parasite invasion throughout the apicomplexans (Fig 8E-8F and S6 Table). The spherical bodies of piroplasm parasites are reportedly analogous to the dense granules of other apicomplexan parasites, which play an essential role in parasite invasion. Truncated copies 11 of spherical body protein 2 was found to be high expressed in attenuated strains, and up-regulation of sbp2t11 is accompanied by a statistically significant reduction in cytoadhesion, suggesting that this protein may play roles associated with erythrocyte infection. In vitro neutralization tests using anti-BcSBP4 peptide antibodies showed a significant inhibitory effect on the infectivity of *B. caballi* merozoites in horse red blood cells [42,52,53]. This is consistent with the reduced invasion efficiency of the strain of AP2-M2 disrupted. We also observed the significantly downregulated expression of genes encoding tubulin, inner membrane complex proteins, and membrane proteins, which may have contributed to the morphological changes observed in ΔBxAP2-M2 merozoites. Among these downregulated genes, 82 were annotated as encoding uncharacterized protein, indicating that they are involved in unknown functions. To investigate whether novel developmental stages or clusters appear in the ΔBxAP2-M2 strain, we also performed single-cell RNA sequencing (scRNA-seq) on the mutant. Compared with the wild-type *B. xinjiangensis*, we did not identify more or fewer clusters in ΔBxAP2-M2 (S5C Fig).

## Discussion

In this study, we provide the first chromosome-level genomic sequence, its annotation and transcriptomic patterns; homogeneous cell subpopulations during asexual development; and an AP2 protein that regulates merozoite development in the causative agent of ovine babesiosis, *B. xinjiangensis*. *Babesia xinjiangensis* can be continuously cultured in sheep red blood cells in vitro. Furthermore, because the genome of its transmission vector (*Hyalomma* spp.) is completely sequenced, *B. xinjiangensis* is an ideal model parasite for research into the mechanisms underlying various parasite features, including the regulation of its periodical development and its transmission. The increasing available genomic resources available for *Babesia* spp., should facilitate the identification of conserved and dispensable pathways that are potential targets for anti-babesiosis drugs or blocking the transmission progression of the parasite from its vector to vertebrate host.

We used the genomic information for this novel species to investigate the phylogenetic relationships between *B. xinjiangensis* and other apicomplexan parasites, its speciation time, the multigene families present that correlate with immune evasion, and its interactions with its host, in a full biological characterization of this parasite. Our analysis confirmed the phylogenetic position of *B. xinjiangensis*. Consistent with these relationships, we noted that the genomic organization of *B. xinjiangensis* is more similar to that of *B. bovis* than to that of any other piroplasm parasite, including in its genome size, GC content, and variant *vesa* gene family, which is responsible for the parasite’s survival in mammalian red blood cells and its escape from its host’s immune response.

The most prominent feature of *B. xinjiangensis* is the presence of gene family duplications. Ninety-eight genes belonging to orthologous groups, which have one or two orthologous genes in other piroplasmid species, have more than three in the *B. xinjiangensis* genome. Sequence-length variants and sequence divergence were also detected in this parasite, indicating that gene family duplication events have occurred during its species speciation and evolution. We identified unique genes and gene families in *B. xinjiangensis* that share no amino acid or nucleotide sequence homologies with those of other apicomplexan species. Although the amino acid sequence similarity in each BxjU family is low, conserved regions and motifs are present. Interestingly, most members of BxjUF1 and BxjUF3 are located in proximate telomeres, subtelomeres, or centromeres. In *Plasmodium* spp., the *var* genes are also located at sub-telomeres and are associated with immune evasion and parasite virulence. In protists, species-specific gene families are sources of novel genes, and are distributed in similar positions in genomes. These genes are usually not conserved across species, but they are related to the environmental adaptation and expressed in response to specific environmental stimuli [6,11,54].

We performed the single-cell transcriptomic sequencing during asynchronously asexual replication of *B. xinjiangensis* and demonstrated the variation in the transcriptional profiles of individual cells. Taking advantage of the single-cell sequencing platform, we not only identified distinct cell populations, but also identified potentially differential functions form one parasite to another. Our understanding of the asexual replication of *Babesia* spp. has been defined by studies of other apicomplexan parasites (e.g., *T. gondii* and *Plasmodium* spp.). A previous study using scRNA-seq characterized the transcription patterns during the asexual development of *B. bovis*, *B. bigemina*, and *B. divergens*, but no gametocytes were observed. A major finding of the present study, based on the analysis of scRNA-seq sequences and additional molecular features, is that at least two cell populations exist in *B. xinjiangensis*. Importantly, our study identified a set of necessary markers that distinguish gametocytes, which will greatly facilitate future studies of its gametocytogenesis mechanisms and the identification of the critical regulators of gametocyte differentiation.

We inferred the replicative-cycle phases of *B. xinjiangensis* using the well-studied markers of *T. gondii* and *Babesia* spp. replicative cycle phases from single cell sequencing and DNA content analysis. We also observed overlaps in the peak expression times of the *B. xinjiangensis* markers in distinct cell states, and reported some new aspects of *Babesia* biology. Some rhoptry proteins are expressed in two replicative-cycle phases, suggesting that overlapping transition boundaries are conserved across Apicomplexa. Smooth transcriptional transitions across the phases of asexual replication were observed among distinct cell states. The asexual development of *Babesia* parasite is a complex process, consisting of DNA replication, the assembly of the red-blood-cell-invasion machinery, the formation of moving junctions, and the time-dependent expression of rhoptry proteins. Strict checkpoints are required to control the orders of these cellular events. We propose that the smooth transcriptional transitions are associated with these checkpoints, although the regulators of them were not identified.

This dataset illustrates the full landscape of *B. xinjiangensis* development within its vertebrate host, provides insights into how these complex cellular events are modulated, and offers potential targets for blocking the asexual replication and gameteocyogenesis of *B. xinjiangensis* to control babesiaosis. Because our analysis examined gametocyte transcriptomes and evaluated gene expression levels, we identified unexpected potential functions of well-studied and uncharacterized genes that were specifically expressed in subpopulations of cells. A possible role of cysteine proteinase in merozoite egress suggests that a conserved egress mechanism acts in multiple developmental stages of the life cycle. More importantly, this global view of gene expression across the asexual life cycle should provide comprehensive strategies to support the investigation of antibabesiosis interventions. Multistage functional molecules may be attractive targets for vaccine development and drug interventions, at the levels of both vertebrate host and tick vector. We examined the dynamic expression of several gene families, including epigenetic markers, invasion-related genes, and AP2s. Most members of the observed gene families shown specific times of peak expression throughout the replication cycle, whereas the transcription factor BxAP2-M2 was constantly expressed throughout our analysis, indicating its important role in the asexual cycle of *Babesia*. Future studies in which these genes are knocked out will provide experimental evidence of the factors that regulate gene transcription, and identify novel roles for these proteins in parasite development.

With genetic analyses, we identified and characterized the AP2 transcription factor BxAP2-M2, which is crucial for *B. xinjiangensis* growth. Disrupting BxAP2-M2 altered the expression of the majority of morphology- and invasion-related genes, reducing the efficiency of host-cell invasion and the morphological changes in *B. xinjiangensis*. Therefore, dissecting the roles and underlying mechanisms of BxAP2-M2 is essential for understanding how AP2s are involved in merozoite growth and invasion. A comparative analysis of the transcriptomes of the parental and mutant strains showed that BxAP2-M2 directly or indirectly regulates the expression of genes at asexual life cycle. The expression profiles of many genes that are normally expressed in asexual replication were disordered in the BxAP2-M2-knockout strain. These included S/M phases markers RON4, RON5, and RON8, which are core components of the invasion machinery conserved across Apicomplexa, moving junction. Unexpectedly, the expression of RON4 was upregulated in the BxAP2-M2-knockout parasite (S4 Table). We speculate that the upregulation of these two RONs complements the downregulation of for RON4, RON5, and RON8. An alternative invasion machinery may exist, composed of homologues of RON4, RON5, and RON8, although its efficiency may be relatively low, reflecting the reduced host-cell invasion by the BxAP2-M2-knockout strain. The downregulated expression of rhoptry proteins caused a severe defect in host-cell invasion. It is also possible that parasite invasion may be completely blocked by disruption of these genes.

BxAP2-M2 is involved in the regulation of multiple genes, but the exact mechanisms of its action are largely unknown. Increasing evidence reveals that the AP2 transcription factors may cooperate with others or interact with epigenetic factors, such as HDAC or GCN5b, to regulate gene expression [55–57]. The expression of BxAP2-M2 does not seem to fluctuate during the cell cycle, as demonstrated by our single-cell data. This suggests that additional regulators are responsible for the time-course expression of BxAP2-M2. Interestingly, the expression levels of three AP2 proteins were altered in the BxAP2-M2 mutant strain (ΔBxAP2-M2), including GWHPERCV003543, GWHPERCV000666, and GWHPERCV001688. To ensure the accurate and precise regulation of gene expression, cross-talk among AP2s and a dynamic cascade network of transcription factors may present the entire life cycle.

In conclusion, in this study, we provide a chromosome-level genome assembly of *B. xinjiangensis*, a valuable resource for understanding the biological features of *Babesia*. This parasite shares a close phylogenetic relationship with *B. bovis*. We also identified unique and core gene families in *B. xinjiangensis* that may be involved in the parasite’s survival, speciation, and immune evasion in its hosts. We traced the heterogeneous development of the asexual replication of this unicellular *Babesia* parasite and characterized the transcriptional variations in a large number of cells, using a single-cell sequencing approach and analysis protocol. One of our major findings was a sub population of cells, characterized by gametocytes markers HAP2 and dynein heavy chain. Leveraging genetic approach, we have demonstrated the role of an AP2 transcription factor, BxAP2-M2, which is constantly and strongly expressed throughout asexual replication. This transcription factor directly or indirectly regulates the expression of genes encoding rhoptry-associated protein, which are responsible for parasite invasion. We believe that identifying the roles of the target genes of BxAP2-M2 could clarify the mechanisms regulating the parasite’s asexual cycle.

## Materials and methods

### Ethics approval

The collection and manipulation of sheep blood samples was approved by the Animal Ethics Committee of the Lanzhou Veterinary Research Institute, Chinese Academy of Agricultural Sciences. All sampling procedures were handled in accordance with the Animal Ethics Procedures and Guidelines of the People’s Republic of China (Permit No. LVRIAEC-2018-001). All the procedures were conducted according to the Ethical Procedures and Guidelines of the People’s Republic of China.

### Parasite culture

*Babesia xinjiangensis* was maintained in vitro using a complete medium consisting of RPMI-1640 medium (CellMax, Beijing, China) supplemented with 20% of fetal bovine serum (FBS; CellMax, Beijing, China) and 7.5% of fresh sheep red blood cells [17]. The medium was refreshed every 48 hours. When the parasitemia reached 20%, parasites were harvested by centrifugation (1,000 × g, 10 minutes, 4°C). Merozoites were then purified using a modification method [58]. Briefly, infected erythrocytes were treated with 0.05% saponin in phosphate-buffered saline (PBS), followed by centrifugation (10,000 × g, 30 minutes, 4°C) to isolate intact merozoites [11].

### Genomic DNA and RNA extraction and sequencing

Genomic DNA was extracted from the purified *B. xinjiangensis* merozoites with the QIAamp DNA Blood Mini Kit (Qiagen, Hilden, Germany), following the manufacturer’s protocol. Long DNA fragments were selected using the BluePippin system (Sage Science, USA). Following end repair, a DNA library was constructed with Oxford Nanopore Technology (ONT) ligation sequencing kit (SQK-LSK109) to generate adapter-ligated long reads and sequenced using two FLOMIN106 flow cells (v9.4.1). The raw FAST5 data was base called using Guppy (v3.2.2). Illumina sequencing data from libraries of genomic DNA containing 400-bp paired-end reads using MGISEQ-2000RS sequencing platform (MGI Tech, Shenzhen, China), used for correction the assembled genome with long reads. To anchor assembled scaffolds onto the chromosomes, we constructed a chromosome conformation capture (Hi-C) library and generated data with MGISEQ-2000RS sequencing platform (MGI Tech, Shenzhen, China). In brief, purified *B. xinjiangensis* merozoites was incubated in 2% formaldehyde for 30 min at 37°C. crosslinking was stopped by adding glycine. Genomic DNA was digested with DpnII and marked with biotin-14-dCTP. The tagged DNA was sheared into ~400 bp fragments and blunt-end repaired and A-tailed. Then, the Hi-C library was sequenced. Total RNA was extracted with the TRIzol Reagent (Cat: 15596018, Invitrogen, USA), according to the manufacturer’s kit instructions. Libraries were constructed and sequenced using with the standard MGISEQ-2000RS platform.

### *De novo* genome assembly

After low-quality reads and bases were removed with the NanoLyse and NanoFilt packages, the ONT reads were utilized to assemble a continuous genomic sequence, as the previously reported pipeline for piroplasms [59]. NECAT (v0.0.1) and Canu (v2.2.2) were used to assemble the ONT reads into draft contigs, which were merged into one genome to improve its contiguity, with the quickmerge package (https://github.com/mahulchak/quickmerge) [60,61]. To improve the accuracy of the draft contigs, three rounds of self-correction with ONT reads and Illumina sequencing reads were performed using minimap2, racon, and NextPolish programs [62–64]. Hi-C data were used to order the contigs and the mis-joins were correct with the ALLHiC package [65]. Whether the *B. xinjiangensis* genome contained Apicomplexa core genes in the apicomplexa_odb10 dataset was examined with Benchmarking Universal Single-Copy Orthologs (BUSCO v5.1.3) [32].

### Genome annotation

A standard pipeline was applied according to the previous reporter by Wang et al. (2023), involving (ⅰ) repeat sequence annotation (the prediction of simple tandem repeats using TRF [v4.09], the identification of de novo repeats with RepeatScout [v1.0.5], and the construction of a homologous alignment with RepeatMasker); (ⅱ) gene prediction with integrated *ab initio* modeling, homology alignment, and transcriptomic data. PASA (v2.3.1) was used to obtain candidate gene models, which were a good source of the training models used in Augustus (v3.3.3) and GlimmerHMM (v3.0.4) [33,66–69]. These trained models were then used to predict the gene structures with Augustus and GlimmerHMM [34]. Additional evidence was obtained by aligning the candidate gene structures against the UniProt Apicomplexa database with Exonerate (v2.4.0) and GeneID (v1.4.5) to incorporate transcriptome evidence [35,70,71]. Illumina RNA-seq reads were aligned against the *B. xinjiangensis* genome with TopHat2 (v2.1.1), and transcripts were generated with Cufflinks (v2.2.1) [72,73]. The combined evidence from ab initio predictions, homology alignments, and transcriptomic data were integrated into a nonredundant gene set with EvidenceModeler (v1.1.1), which was then further manually curated [74].

### Orthologous group identification and gene family analysis

The predicted protein-coding genes of 18 Apicomplexan species or subspecies, including *B. xinjiangensis* (GWHERCV00000000 and GWHERCV00000000.2), *Babesia bovis* (GCA_000165395.2), *B. m. lintanensis* (GWHBEID00000000), *B. m. hebeiensis* (GWHBEIF00000000), *B. bigemina* (GCF_000981445.1), *B. microti* (GCF_000691945.2), *Babesia ovata* (GCA_002897235.1), *Theileria equi* (GCA_000342415.1), *Theileria orientalis* (GCA_000740895.1), *Theileria annulata* (GCF_000003225.3), *Theileria parva* (GCA_000165365.1), *Plasmodium vivax* (GCA_000002415.2), *Plasmodium yoelii* (GCA_900002385.2), *Plasmodium falciparum* (GCA_000002765.3), *To. Gondii* (GCA_000006565.2), *C. parvum* (GCA_000165345.1), *E. tenella* (GCA_000499545.1), *N. caninum* (GCA_000208865.2), and an outgroup species of *Te. Thermophila* (GCA_000189635.1), were obtained from the National Center for Biotechnology Information (NCBI), PlasmoDB, and China National Center for Bioinformation (CNCB). Orthologous groups across these species were identified with OrthoFinder (v2.5.4), an effective tool for clustering orthologues in multispecies datasets, using the DIAMOND program to align the amino acid sequences with the inflation index set at 1.5 to balance sensitivity and specificity [75]. The orthologous groups identified were then analyzed for gene family expansions and contractions with CAFE (v2.0), which uses a computational approach to detect evolutionary shifts in the size of gene families [76].

### Phylogenetic analysis and estimation of divergence times

A total of 337 single-copy orthologous genes were identified across 18 Apicomplexan species, when *Te. thermophila* was used as the outgroup. Using this dataset, a maximum likelihood phylogenetic tree was constructed in RAxML with the LG + F + R6 model [77]. The divergence times were estimated with MCMCTree, calibrated with three known divergence points [78]. The phylogenetic trees were visualized and labeled with ITOL (http://itol2.embl.de/upload.cgi).

### Identification of gene families

To identify the genes associated with transcriptional regulation, motility, epigenetic markers, and immune evasion in *B. xinjiangensis* genome, we used a combination of BLASTP and the Hidden Markov Model (HMM). The protein sequences of these functionally annotated genes were aligned with MUSCLE [79]. HMM models were generated with HMMER, allowing a precise search for motifs within the *B. xinjiangensis* genome in order to identify proteins with conserved domains and motifs. Custom scripts were used to annotate the identified genes, visualizing their distribution and characteristics across the genome.

### scRNA-seq and data analysis

When the parasitemia of *in vitro*-cultured *B. xinjiangensis* reached 20%, a total of 20,000 cells were washed three times with cold PBS and then loaded into the Chromium Chip B. scRNA-seq libraries were sequenced with the Chromium Single Cell 3’ platform (10 × Genomics). The raw scRNA-seq data were aligned with the *B. xinjiangensis* genome with the Cell Ranger pipeline (v3.1) to generate gene expression matrices. The data were then analyzed with the Seurat package (v5.0) for clustering and dimensionality reduction [80]. Uniform manifold approximation and projection (UMAP) was used to identify distinct cell populations, and principal component analysis (PCA) was performed to determine the population structure based on gene expression levels. Differentially expressed genes (DEGs) were identified with the criteria of fold change > 1.5 and an adjusted P-value < 0.01, to identify the cell-type-specific transcriptional profiles related to essential processes, such as metabolism, chromosome replication, and organelle biogenesis. A further trajectory analysis was conducted with Monocle 2, using pseudotime to model the developmental transitions from the early merozoite stages to pre-egress and gametocyte-like stages. In this way, we established the progression of gene expression changes throughout the asexual replication cycle [81,82]. The analysis R code is referenced the scripts with minor modifications (GitHub at: https://github.com/umbibio/scBabesiaAtlases and https://doi.org/10.1038/s41587-019-0206-z) [28,83].

### Construction of BxAP2-M2 knockout and complementation strain

Homologous recombination approach was used to construct a parasite strain in which the BxAP2-M2 gene was disrupted. To construct the BxAP2-M2 gene disruption vector (pΔBxAP2-M2), two sequences of BxAP2-M, the 5’ and 3’ untranslated regions (UTRs), in the KO-AP2-M plasmid were replaced with BxAP2-M2 5’ and 3’ UTRs, respectively [82]. To construct the plasmid complementing BxAP2-M2 (pcBxAP2-M2), the *ap2-m* open reading frame (ORF) sequence was replaced with the full-length of BxAP2-M2-encoding gene and FLAG tag sequence. *Babesia xinjiangensis* merozoites were transformed with the pΔBxAP2-M2 plasmid (20 µg). At 24 h after transfection, the medium was supplemented with 10 μg/ml of blasticidin S (BSD) to select for parasites resistance to BSD drug. Clonal parasites of the BxAP2-M2-gene-disrupted strain (ΔBxAP2-M2) were confirmed with PCR, Western blotting, and indirect immunofluorescence assays. Furthermore, ΔBxAP2-M2 merozoites were transfected with the pcBxAP2-M2:C plasmid (20 µg) to generate a complement strain (ΔBxAP2-M2:C), which were selected with bleomycin (200 μg/ml). The clonal strain of ΔBxAP2-M2:C was confirmed with PCR and indirect immunofluorescence (S4 Fig). All the primers used are listed in S7 Table.

### Western blotting

Red blood cells (RBCs) infected with parasites were collected by centrifugation (1,500 × g for 10 min at 4°C) and the merozoites were released from the erythrocytes using a red blood cell lysis buffer (containing 1.55 M NH₄Cl, 0.1 M KHCO₃, and 1 mM Na₂EDTA), which was obtained from Solarbio Science & Technology Co., Ltd. in Beijing, China. The merozoite pellets were treated with radioimmunoprecipitation assay (RIPA) lysis buffer containing 1 × Protease and Phosphatase Inhibitor Cocktail. The insoluble fraction of the parasite lysate was removed by centrifugation at 12,000 × g for 10 min at 4°C. The supernatants, containing 1 × Protein Loading Buffer, was boiled for 10 min. The samples were separated with sodium dodecyl sulfate-polyacrylamide gel electrophoresis (SDS-PAGE) and transferred to polyvinylidene fluoride membranes (MILLIPORE, Billerica, MA, USA). Anti-β-actin (Proteintech # 81115–1-RR), anti-FLAG antibodies (Proteintech # 20543–1-AP), and anti-HA antibodies (Proteintech # 66006–2-Ig) were reacted with the proteins on the membranes, as previously reported, and then secondary antibodies conjugated a peroxidase were used to recognize the relevant primary antibodies. The signals were developed with the Enhanced Chemiluminescence Detection Kit (Proteintech, Wuhan, China; PK10001) and visualized the ChemiDoc XRS+ (Bio-Rad, USA).

### RNA sequencing

Total RNA was extracted form *B. xinjiangensis* merozoites and ΔBxAP2-M2 cultured in vitro and sequenced. When parasitemia reached 20%, the cultures of *B. xinjiangensis* and ΔBxAP2-M2 were harvested by centrifugation at 1,500 × g for 10 min at 4°C. The intraerythrocytic merozoites were released with red blood cell lysis buffer and collected with centrifugation at 12,000 × g for 10 min at 4°C. The total RNA was extracted from the merozoites with the RNeasy Mini Kit (Qiagen, MD, USA), and its quality was assessed on an Agilent 2100 Bioanalyzer. RNA-Seq libraries were prepared from three biological replicates using the Illumina TruSeq Stranded mRNA Library Prep Kit and sequenced on the MGISEQ-2000 RS platform. After low-quality reads/bases and adapter sequences were removed, RNA-seq data were mapped to the reference genome with TopHat2. The gene expression levels were quantified in fragments per kilobase of transcript per million mapped fragments units (FPKM) with Cufflinks. DESeq2 (v1.20.0) was used to identify the differential expression genes (DEGs) with threshold values: *P*-value < 0.05 and absolute fold change > 1.5.

### Real-time quantitative PCR

Significantly DEGs were further confirmed with real time quantitative PCR (RT-qPCR) on the Mx3005P system (Agilent, Canada). The reaction parameters were: initial denaturation at 95°C for 5 min, 40 cycles of 95°C for 5 s and 60°C for 30 s. the glyceraldehyde-3-phosphate dehydrogenase (GAPDH) gene was used as the internal control. Three biological replicates were performed for each gene. The primer pairs used are listed in S6 Table.

### Proteomic analysis

The level of protein expression was evaluated with label-free liquid chromatography-tandem mass spectrometry (LC-MS/MS). Merozoites were collected from *B. xinjiangensis* and ΔBxAP2-M2 cultures and purified as described in previous section. The merozoite pellets were treated with radioimmunoprecipitation assay (RIPA) lysis buffer containing 1 × Protease and Phosphatase Inhibitor Cocktail. The insoluble fraction of parasite lysate was removed by centrifugation at 12,000 × g for 10 min at 4°C. The supernatant was digested with trypsin, and the peptides were analyzed with LC-MS/MS on a Q-Exactive Plus Hybrid Quadripole-Orbitrap Mass Spectrometer coupled to a Dionex UltiMate 3000 UPLC system. The data were analyzed with the MaxQuant software (v1.6.14) to identify and quantify the proteins. The annotated *B. xinjiangensis* protein sequences were used to construct the reference database for proteomic analysis.

### Invasion and egress assays

The merozoites invasion efficiency was measured as previously described [84]. Briefly, merozoites were isolated from 1 × 10^9^ infected red blood cells by incubation in RPMI-1640 supplemented with 10% DMSO for 10 min. The released merozoites were then resuspended into complete medium containing 5% fresh sheep red blood cells and cultured at 37°C for 6 h. to quantify invasion efficiency, parasitemia was calculated by determining the infection rate in 2,000 red blood cells using Giemsa-stained smears. All experiments were performed in triplicate with independent biological replicates. Parasitemia (%) = (number of infected RBCs in 2000 RBCs/ 2,000 total RBCs counted) × 100%. Invasion inhibition (%) = (mean parasitemia in control group – mean parasitemia in experimental group)/ mean parasitemia in control group × 100%.

Free merozoites were removed by centrifugation (500 × g, 5 min, 4°C). After 12 h of merozoites invasion, the percentage of single piriform and paired piriform merozoites were determined in 2,000 red blood cells (*n* = 3 biological replicates). 2 μM calimycin was added to the cultures, which were then vigorously shaken (200 rpm) for 3 h. Parasitemia was calculated by determining the infection rate in 2,000 red blood cells and was used to calculate the efficiency of egress (*n* = 3 biological replicates). Egress efficiency (%) = (mean parasitemia in control group – mean parasitemia in experimental group)/ mean parasitemia in control group × 100%.

### Immunostaining

When parasitemia reached 20%, the cultures were washed three times with phosphate buffered saline (PBS) and RBCs were fixed on microscope slides with 4% paraformaldehyde for 10 min. after the slides were washed three times with PBS, cells were permeabilized with 0.5% Triton X-100, and then blocked with 1% bovine serum albumin (BSA) in PBS at 37°C for 1 h. The cells were then incubated overnight at 4°C with either a primary mouse anti-HA or mouse anti-FLAG antibody diluted with blocking solution. After the microscope slides were washed three times with PBS, they were incubated with a Fluorescein (FITC)-conjugated donkey anti-mouse IgG. The microscope slides were mounted with Prolong Gold Antifade Mountant with DNA Stain DAPI (Invitrogen, CA, USA) and images were captured with an Olympus Fluoview FV1000 confocal microscope.

## Supporting information

S1 Fig Genomic features of *Babesia xinjiangensis.* (A) Completeness of the *Babesia xinjiangensis* genome and predicted protein-coding genes. (B) Speciation time of *Babesia xinjiangensis*. Species dates were estimated from 321 single-copy orthologues (2,929,403 amino acids) across the reference genomes of 18 Apicomplexa parasites and an outgroup species. (C) Genomic synteny of *B. xinjiangensis* with *B. duncani*, *B. bovis*, and *B. motasi hebeiensis*.
(TIF)

S2 Fig Distributions and conserved motifs of unique gene families in *Babesia xinjiangensis* genome. (A) Distributions of unique gene families in *B. xinjiangensis* genome. B, C, and D showing the conserved motifs in BxjUF1, BxjUF2, and BxjUF3.
(TIF)

S3 Fig Characterization of BxAP2-M2. (A) Position of AP2 domains in protein structure of BxAP2-M2. (B) Predicted AP2 domain in Piroplasm parasites. (C) Phylogenetic analysis of BxAP2-M2 and its homologues in other piroplasm parasites. (D) Predicted protein structure of BxAP2-M2.
(TIF)

S4 Fig Complemented strain ΔBxAP2-M2:C confirmed with PCR and immunofluorescence assay. (A) Diagram showing strategy of constructing a complement strain. (B) Complemented strain confirmed with PCR assay. (C) ΔBxAP2-M2:C strain validated with immunofluorescence assay.
(TIF)

S5 Fig Analysis of significantly down-regulated genes and comparison of cell clusters. (A) Gene Ontology (GO) analysis of the significantly down regulated genes. (B) Pathway analysis based on the Kyoto Encyclopedia of Genes and Genomes (KEGG) for the significantly down regulated genes. (C) Cell cluster comparison between *B. xinjiangensis* and ΔBxAP2-M2 using single-cell transcriptional sequencing (scRNA-seq).
(TIF)

S1 Table Genomic features and gene families in *B. xinjiangensis.* (XLSX)

S2 Table Cluster markers and pseudotime differential genes of asexual replication in *B. xinjiangensis.* (XLSX)

S3 Table Invasion and egress assaysDifferentially expressed genes in BxAP2-M2-disrupted strain ΔBxAP2-M2. (XLSX)

S4 Table Differential expressed genes in BxAP2-M2 disrupted strain. (XLSX)

S5 Table Significantly differential expressed genes confirmed with real time quantitative PCR. (XLSX)

S6 Table Different expressed proteins in BxAP2-M2 disrupted strain ΔBxAP2-M2. (XLSX)

S7 Table Primer sequences used in this study. (XLSX)

## References

[1] Homer MJ, Aguilar-Delfin I, Telford SR 3rd, Krause PJ, Persing DH. Babesiosis. Clin Microbiol Rev. 2000;13(3):451–69. doi: 10.1128/CMR.13.3.451 10885987 PMC88943

[2] Kivaria FM. Estimated direct economic costs associated with tick-borne diseases on cattle in Tanzania. Trop Anim Health Prod. 2006;38(4):291–9. doi: 10.1007/s11250-006-4181-2 17137131

[3] Bock R, Jackson L, de Vos A, Jorgensen W. Babesiosis of cattle. Parasitology. 2004;129 Suppl:S247-69. doi: 10.1017/s0031182004005190 15938514

[4] He L, Bastos RG, Sun Y, Hua G, Guan G, Zhao J, et al. Babesiosis as a potential threat for bovine production in China. Parasit Vectors. 2021;14(1):460. doi: 10.1186/s13071-021-04948-3 34493328 PMC8425137

[5] Scott JD, Scott CM. Human Babesiosis Caused by Babesia duncani Has Widespread Distribution across Canada. Healthcare (Basel). 2018;6(2):49. doi: 10.3390/healthcare6020049 29772759 PMC6023460

[6] Wang J, Chen K, Yang J, Zhang S, Li Y, Liu G, et al. Comparative genomic analysis of Babesia duncani responsible for human babesiosis. BMC Biol. 2022;20(1):153. doi: 10.1186/s12915-022-01361-9 35790982 PMC9258201

[7] Kim TY, Kim SY, Seo J, Lee HI, Lee W, Kim H. Detection of US and Kobe‐type Babesia microti in ticks collected from small mammals of the Republic of Korea. Entomological Research. 2024;54(2). doi: 10.1111/1748-5967.12708

[8] Jia N, Zheng Y-C, Jiang J-F, Jiang R-R, Jiang B-G, Wei R, et al. Human Babesiosis Caused by a Babesia crassa-Like Pathogen: A Case Series. Clin Infect Dis. 2018;67(7):1110–9. doi: 10.1093/cid/ciy212 29538646

[9] Hong S-H, Kim S-Y, Song BG, Rho J-R, Cho CR, Kim C-N, et al. Detection and characterization of an emerging type of Babesia sp. similar to Babesia motasi for the first case of human babesiosis and ticks in Korea. Emerg Microbes Infect. 2019;8(1):869–78. doi: 10.1080/22221751.2019.1622997 31179860 PMC6566668

[10] Wang J, Zhang S, Yang J, Liu J, Zhang D, Li Y, et al. Babesia divergens in human in Gansu province, China. Emerg Microbes Infect. 2019;8(1):959–61. doi: 10.1080/22221751.2019.1635431 31244397 PMC6598508

[11] Wang J, Chen K, Ren Q, Zhang S, Yang J, Wang Y, et al. Comparative genomics reveals unique features of two Babesia motasi subspecies: Babesia motasi lintanensis and Babesia motasi hebeiensis. Int J Parasitol. 2023;53(5–6):265–83. doi: 10.1016/j.ijpara.2023.02.005 37004737

[12] Wang X, Wang J, Liu J, Liu A, He X, Xiang Q, et al. Insights into the phylogenetic relationships and drug targets of Babesia isolates infective to small ruminants from the mitochondrial genomes. Parasit Vectors. 2020;13(1):378. doi: 10.1186/s13071-020-04250-8 32727571 PMC7391622

[13] Sevinc F, Zhou M, Cao S, Ceylan O, Aydin MF, Sevinc M, et al. Haemoparasitic agents associated with ovine babesiosis: A possible negative interaction between Babesia ovis and Theileria ovis. Vet Parasitol. 2018;252:143–7. doi: 10.1016/j.vetpar.2018.02.013 29559137

[14] Jalovecka M, Sojka D, Ascencio M, Schnittger L. Babesia Life Cycle - When Phylogeny Meets Biology. Trends Parasitol. 2019;35(5):356–68. doi: 10.1016/j.pt.2019.01.007 30733093

[15] White MW, Suvorova ES. Apicomplexa Cell Cycles: Something Old, Borrowed, Lost, and New. Trends Parasitol. 2018;34(9):759–71. doi: 10.1016/j.pt.2018.07.006 30078701 PMC6157590

[16] Jalovecka M, Hajdusek O, Sojka D, Kopacek P, Malandrin L. The Complexity of Piroplasms Life Cycles. Front Cell Infect Microbiol. 2018;8:248. doi: 10.3389/fcimb.2018.00248 30083518 PMC6065256

[17] Guan G, Ma M, Liu A, Du P, Ren Q, Li Y, et al. Continuous in vitro cultivation of a recently identified Babesia that infects small ruminants in China. Vet Parasitol. 2012;187(3–4):371–8. doi: 10.1016/j.vetpar.2012.02.006 22386948

[18] Guan G, Moreau E, Brisseau N, Luo J, Yin H, Chauvin A. Determination of erythrocyte susceptibility of Chinese sheep (Tan mutton breed) and French sheep (Vendéen breed) to Babesia sp. BQ1 (Lintan) by in vitro culture. Vet Parasitol. 2010;170(1–2):37–43. doi: 10.1016/j.vetpar.2010.02.006 20223592

[19] Abraham A, Brasov I, Thekkiniath J, Kilian N, Lawres L, Gao R, et al. Establishment of a continuous in vitro culture of Babesia duncani in human erythrocytes reveals unusually high tolerance to recommended therapies. J Biol Chem. 2018;293(52):19974–81. doi: 10.1074/jbc.AC118.005771 30463941 PMC6311517

[20] Grande N, Precigout E, Ancelin ML, Moubri K, Carcy B, Lemesre JL, et al. Continuous in vitro culture of Babesia divergens in a serum-free medium. Parasitology. 1997;115 ( Pt 1):81–9. doi: 10.1017/s0031182097008937 9226955

[21] Li D, Wang L, Guan X, Wang S, Liu Q, Chen F, et al. Establishment of Continuous In Vitro Culture of Babesia gibsoni by Using VP-SFM Medium with Low-Concentration Serum. Microbiol Spectr. 2023;11(3):e0025823. doi: 10.1128/spectrum.00258-23 37158742 PMC10269504

[22] Pedroni MJ, Sondgeroth KS, Gallego-Lopez GM, Echaide I, Lau AOT. Comparative transcriptome analysis of geographically distinct virulent and attenuated Babesia bovis strains reveals similar gene expression changes through attenuation. BMC Genomics. 2013;14:763. doi: 10.1186/1471-2164-14-763 24195453 PMC3826834

[23] Santamaria RM, Estrada K, López ME, Rojas E, Martínez G, Alcalá Y, et al. Comparative Transcriptome Analysis of Babesia bigemina Attenuated Vaccine and Virulent Strains of Mexican Origin. Vaccines (Basel). 2024;12(3):309. doi: 10.3390/vaccines12030309 38543943 PMC10975891

[24] González LM, Estrada K, Grande R, Jiménez-Jacinto V, Vega-Alvarado L, Sevilla E, et al. Comparative and functional genomics of the protozoan parasite Babesia divergens highlighting the invasion and egress processes. PLoS Negl Trop Dis. 2019;13(8):e0007680. doi: 10.1371/journal.pntd.0007680 31425518 PMC6715253

[25] Howick VM, Russell AJC, Andrews T, Heaton H, Reid AJ, Natarajan K, et al. The Malaria Cell Atlas: Single parasite transcriptomes across the complete Plasmodium life cycle. Science. 2019;365(6455):eaaw2619. doi: 10.1126/science.aaw2619 31439762 PMC7056351

[26] Poran A, Nötzel C, Aly O, Mencia-Trinchant N, Harris CT, Guzman ML, et al. Single-cell RNA sequencing reveals a signature of sexual commitment in malaria parasites. Nature. 2017;551(7678):95–9. doi: 10.1038/nature24280 29094698 PMC6055935

[27] Xue Y, Theisen TC, Rastogi S, Ferrel A, Quake SR, Boothroyd JC. A single-parasite transcriptional atlas of Toxoplasma Gondii reveals novel control of antigen expression. Elife. 2020;9:e54129. doi: 10.7554/eLife.54129 32065584 PMC7180058

[28] Rezvani Y, Keroack CD, Elsworth B, Arriojas A, Gubbels M-J, Duraisingh MT, et al. Comparative single-cell transcriptional atlases of Babesia species reveal conserved and species-specific expression profiles. PLoS Biol. 2022;20(9):e3001816. doi: 10.1371/journal.pbio.3001816 36137068 PMC9531838

[29] Guan G, Ma M, Moreau E, Liu J, Lu B, Bai Q, et al. A new ovine Babesia species transmitted by Hyalomma anatolicum anatolicum. Exp Parasitol. 2009;122(4):261–7. doi: 10.1016/j.exppara.2009.05.001 19460377

[30] Guan G, Liu J, Liu A, Li Y, Niu Q, Gao J, et al. A member of the HSP90 family from ovine Babesia in China: molecular characterization, phylogenetic analysis and antigenicity. Parasitology. 2015;142(11):1387–97. doi: 10.1017/S0031182015000797 26156495

[31] Wang J, Chai Y, Yang J, Chen K, Liu G, Luo J, et al. Insight into Hyalomma anatolicum biology by comparative genomics analyses. Int J Parasitol. 2024;54(3–4):157–70. doi: 10.1016/j.ijpara.2023.09.003 37858900

[32] Manni M, Berkeley MR, Seppey M, Simão FA, Zdobnov EM. BUSCO Update: Novel and Streamlined Workflows along with Broader and Deeper Phylogenetic Coverage for Scoring of Eukaryotic, Prokaryotic, and Viral Genomes. Mol Biol Evol. 2021;38(10):4647–54. doi: 10.1093/molbev/msab199 34320186 PMC8476166

[33] Majoros WH, Pertea M, Salzberg SL. TigrScan and GlimmerHMM: two open source ab initio eukaryotic gene-finders. Bioinformatics. 2004;20(16):2878–9. doi: 10.1093/bioinformatics/bth315 15145805

[34] Stanke M, Morgenstern B. AUGUSTUS: a web server for gene prediction in eukaryotes that allows user-defined constraints. Nucleic Acids Res. 2005;33(Web Server issue):W465-7. doi: 10.1093/nar/gki458 15980513 PMC1160219

[35] Parra G, Blanco E, Guigó R. GeneID in Drosophila. Genome Res. 2000;10(4):511–5. doi: 10.1101/gr.10.4.511 10779490 PMC310871

[36] Douzery EJP, Snell EA, Bapteste E, Delsuc F, Philippe H. The timing of eukaryotic evolution: does a relaxed molecular clock reconcile proteins and fossils?. Proc Natl Acad Sci U S A. 2004;101(43):15386–91. doi: 10.1073/pnas.0403984101 15494441 PMC524432

[37] Parfrey LW, Lahr DJG, Knoll AH, Katz LA. Estimating the timing of early eukaryotic diversification with multigene molecular clocks. Proc Natl Acad Sci U S A. 2011;108(33):13624–9. doi: 10.1073/pnas.1110633108 21810989 PMC3158185

[38] Otto TD, Rayner JC, Böhme U, Pain A, Spottiswoode N, Sanders M, et al. Genome sequencing of chimpanzee malaria parasites reveals possible pathways of adaptation to human hosts. Nat Commun. 2014;5:4754. doi: 10.1038/ncomms5754 25203297 PMC4166903

[39] Reyes RA, Raghavan SSR, Hurlburt NK, Introini V, Bol S, Kana IH, et al. Broadly inhibitory antibodies to severe malaria virulence proteins. Nature. 2024;636(8041):182–9. doi: 10.1038/s41586-024-08220-3 39567685 PMC12338075

[40] Bartholdson SJ, Bustamante LY, Crosnier C, Johnson S, Lea S, Rayner JC, et al. Semaphorin-7A is an erythrocyte receptor for P. falciparum merozoite-specific TRAP homolog, MTRAP. PLoS Pathog. 2012;8(11):e1003031. doi: 10.1371/journal.ppat.1003031 23166499 PMC3499583

[41] Satchwell TJ. Erythrocyte invasion receptors for Plasmodium falciparum: new and old. Transfus Med. 2016;26(2):77–88. doi: 10.1111/tme.12280 26862042

[42] Gallego-Lopez GM, Lau AOT, O’Connor RM, Ueti MW, Cooke BM, Laughery JM, et al. Up-regulated expression of spherical body protein 2 truncated copy 11 in Babesia bovis is associated with reduced cytoadhesion to vascular endothelial cells. Int J Parasitol. 2019;49(2):127–37. doi: 10.1016/j.ijpara.2018.05.015 30367864

[43] Mosqueda J, Hernandez-Silva DJ, Ueti MW, Cruz-Reséndiz A, Marquez-Cervantez R, Valdez-Espinoza UM, et al. Spherical Body Protein 4 from Babesia bigemina: A Novel Gene That Contains Conserved B-Cell Epitopes and Induces Cross-Reactive Neutralizing Antibodies in Babesia ovata. Pathogens. 2023;12(3):495. doi: 10.3390/pathogens12030495 36986418 PMC10051436

[44] Alexander DL, Mital J, Ward GE, Bradley P, Boothroyd JC. Identification of the moving junction complex of Toxoplasma gondii: a collaboration between distinct secretory organelles. PLoS Pathog. 2005;1(2):e17. doi: 10.1371/journal.ppat.0010017 16244709 PMC1262624

[45] Hussein HE, Bastos RG, Schneider DA, Johnson WC, Adham FK, Davis WC, et al. The Babesia bovis hap2 gene is not required for blood stage replication, but expressed upon in vitro sexual stage induction. PLoS Negl Trop Dis. 2017;11(10):e0005965. doi: 10.1371/journal.pntd.0005965 28985216 PMC5646870

[46] Hussein HE, Johnson WC, Taus NS, Ueti MW. Expression of sex-specific molecular markers by Babesia bovis gametes. Parasit Vectors. 2024;17(1):75. doi: 10.1186/s13071-024-06185-w 38374075 PMC10877833

[47] Mohammed M, Dziedziech A, Macedo D, Huppertz F, Veith Y, Postel Z, et al. Single-cell transcriptomics reveal transcriptional programs underlying male and female cell fate during Plasmodium falciparum gametocytogenesis. Nat Commun. 2024;15(1):7177. doi: 10.1038/s41467-024-51201-3 39187486 PMC11347709

[48] Real E, Howick VM, Dahalan FA, Witmer K, Cudini J, Andradi-Brown C, et al. A single-cell atlas of Plasmodium falciparum transmission through the mosquito. Nat Commun. 2021;12(1):3196. doi: 10.1038/s41467-021-23434-z 34045457 PMC8159942

[49] Shang X, Wang C, Fan Y, Guo G, Wang F, Zhao Y, et al. Genome-wide landscape of ApiAP2 transcription factors reveals a heterochromatin-associated regulatory network during Plasmodium falciparum blood-stage development. Nucleic Acids Res. 2022;50(6):3413–31. doi: 10.1093/nar/gkac176 35288749 PMC8989538

[50] Modrzynska K, Pfander C, Chappell L, Yu L, Suarez C, Dundas K, et al. A Knockout Screen of ApiAP2 Genes Reveals Networks of Interacting Transcriptional Regulators Controlling the Plasmodium Life Cycle. Cell Host Microbe. 2017;21(1):11–22. doi: 10.1016/j.chom.2016.12.003 28081440 PMC5241200

[51] Zhang C, Li Z, Cui H, Jiang Y, Yang Z, Wang X, et al. Systematic CRISPR-Cas9-Mediated Modifications of Plasmodium yoelii ApiAP2 Genes Reveal Functional Insights into Parasite Development. mBio. 2017;8(6):e01986-17. doi: 10.1128/mBio.01986-17 29233900 PMC5727417

[52] Mahmoud MS, Kandil OM, Abu El-Ezz NT, Hendawy SHM, Elsawy BSM, Knowles DP, et al. Identification and antigenicity of the Babesia caballi spherical body protein 4 (SBP4). Parasit Vectors. 2020;13(1):369. doi: 10.1186/s13071-020-04241-9 32698835 PMC7376649

[53] Gallego-Lopez GM, Lau AOT, Brown WC, Johnson WC, Ueti MW, Suarez CE. Spherical Body Protein 2 truncated copy 11 as a specific Babesia bovis attenuation marker. Parasit Vectors. 2018;11(1):169. doi: 10.1186/s13071-018-2782-z 29530085 PMC5848574

[54] Xiong J, Yang W, Chen K, Jiang C, Ma Y, Chai X, et al. Hidden genomic evolution in a morphospecies-The landscape of rapidly evolving genes in Tetrahymena. PLoS Biol. 2019;17(6):e3000294. doi: 10.1371/journal.pbio.3000294 31158217 PMC6564038

[55] Wang J-L, Li T-T, Zhang N-Z, Wang M, Sun L-X, Zhang Z-W, et al. The transcription factor AP2XI-2 is a key negative regulator of Toxoplasma gondii merogony. Nat Commun. 2024;15(1):793. doi: 10.1038/s41467-024-44967-z 38278808 PMC10817966

[56] Wang J, Chai Y, Yang J, Ye Y, Luo J, Yin H, et al. Dissecting the role of transcription factor AP2-M in Babesia asexual replication. FASEB J. 2024;38(20):e70119. doi: 10.1096/fj.202400127RRR 39441647

[57] Wang J, Dixon SE, Ting L-M, Liu T-K, Jeffers V, Croken MM, et al. Lysine acetyltransferase GCN5b interacts with AP2 factors and is required for Toxoplasma gondii proliferation. PLoS Pathog. 2014;10(1):e1003830. doi: 10.1371/journal.ppat.1003830 24391497 PMC3879359

[58] Guan G, Moreau E, Liu J, Ma M, Rogniaux H, Liu A, et al. BQP35 is a novel member of the intrinsically unstructured protein (IUP) family which is a potential antigen for the sero-diagnosis of Babesia sp. BQ1 (Lintan) infection. Vet Parasitol. 2012;187(3–4):421–30. doi: 10.1016/j.vetpar.2012.01.021 22317784

[59] Wang J, Chen K, Ren Q, Zhang Y, Liu J, Wang G, et al. Systematic Comparison of the Performances of De Novo Genome Assemblers for Oxford Nanopore Technology Reads From Piroplasm. Front Cell Infect Microbiol. 2021;11:696669. doi: 10.3389/fcimb.2021.696669 34485177 PMC8415751

[60] Chen Y, Nie F, Xie S-Q, Zheng Y-F, Dai Q, Bray T, et al. Efficient assembly of nanopore reads via highly accurate and intact error correction. Nat Commun. 2021;12(1):60. doi: 10.1038/s41467-020-20236-7 33397900 PMC7782737

[61] Koren S, Walenz BP, Berlin K, Miller JR, Bergman NH, Phillippy AM. Canu: scalable and accurate long-read assembly via adaptive k-mer weighting and repeat separation. Genome Res. 2017;27(5):722–36. doi: 10.1101/gr.215087.116 28298431 PMC5411767

[62] Li H. Minimap2: pairwise alignment for nucleotide sequences. Bioinformatics. 2018;34(18):3094–100. doi: 10.1093/bioinformatics/bty191 29750242 PMC6137996

[63] Vaser R, Sović I, Nagarajan N, Šikić M. Fast and accurate de novo genome assembly from long uncorrected reads. Genome Res. 2017;27(5):737–46. doi: 10.1101/gr.214270.116 28100585 PMC5411768

[64] Hu J, Fan J, Sun Z, Liu S. NextPolish: a fast and efficient genome polishing tool for long-read assembly. Bioinformatics. 2020;36(7):2253–5. doi: 10.1093/bioinformatics/btz891 31778144

[65] Zhang X, Zhang S, Zhao Q, Ming R, Tang H. Assembly of allele-aware, chromosomal-scale autopolyploid genomes based on Hi-C data. Nat Plants. 2019;5(8):833–45. doi: 10.1038/s41477-019-0487-8 31383970

[66] Benson G. Tandem repeats finder: a program to analyze DNA sequences. Nucleic Acids Res. 1999;27(2):573–80. doi: 10.1093/nar/27.2.573 9862982 PMC148217

[67] Price AL, Jones NC, Pevzner PA. De novo identification of repeat families in large genomes. Bioinformatics. 2005;21 Suppl 1:i351-8. doi: 10.1093/bioinformatics/bti1018 15961478

[68] Flynn JM, Hubley R, Goubert C, Rosen J, Clark AG, Feschotte C, et al. RepeatModeler2 for automated genomic discovery of transposable element families. Proc Natl Acad Sci U S A. 2020;117(17):9451–7. doi: 10.1073/pnas.1921046117 32300014 PMC7196820

[69] Haas BJ, Delcher AL, Mount SM, Wortman JR, Smith RK Jr, Hannick LI, et al. Improving the Arabidopsis genome annotation using maximal transcript alignment assemblies. Nucleic Acids Res. 2003;31(19):5654–66. doi: 10.1093/nar/gkg770 14500829 PMC206470

[70] Slater GSC, Birney E. Automated generation of heuristics for biological sequence comparison. BMC Bioinformatics. 2005;6:31. doi: 10.1186/1471-2105-6-31 15713233 PMC553969

[71] Huang X, Adams MD, Zhou H, Kerlavage AR. A tool for analyzing and annotating genomic sequences. Genomics. 1997;46(1):37–45. doi: 10.1006/geno.1997.4984 9403056

[72] Kim D, Pertea G, Trapnell C, Pimentel H, Kelley R, Salzberg SL. TopHat2: accurate alignment of transcriptomes in the presence of insertions, deletions and gene fusions. Genome Biol. 2013;14(4):R36. doi: 10.1186/gb-2013-14-4-r36 23618408 PMC4053844

[73] Roberts A, Pimentel H, Trapnell C, Pachter L. Identification of novel transcripts in annotated genomes using RNA-Seq. Bioinformatics. 2011;27(17):2325–9. doi: 10.1093/bioinformatics/btr355 21697122

[74] Haas BJ, Salzberg SL, Zhu W, Pertea M, Allen JE, Orvis J, et al. Automated eukaryotic gene structure annotation using EVidenceModeler and the Program to Assemble Spliced Alignments. Genome Biol. 2008;9(1):R7. doi: 10.1186/gb-2008-9-1-r7 18190707 PMC2395244

[75] Emms DM, Kelly S. OrthoFinder: solving fundamental biases in whole genome comparisons dramatically improves orthogroup inference accuracy. Genome Biol. 2015;16(1):157. doi: 10.1186/s13059-015-0721-2 26243257 PMC4531804

[76] Han MV, Thomas GWC, Lugo-Martinez J, Hahn MW. Estimating gene gain and loss rates in the presence of error in genome assembly and annotation using CAFE 3. Mol Biol Evol. 2013;30(8):1987–97. doi: 10.1093/molbev/mst100 23709260

[77] Stamatakis A. RAxML version 8: a tool for phylogenetic analysis and post-analysis of large phylogenies. Bioinformatics. 2014;30(9):1312–3. doi: 10.1093/bioinformatics/btu033 24451623 PMC3998144

[78] dos Reis M, Donoghue PCJ, Yang Z. Bayesian molecular clock dating of species divergences in the genomics era. Nat Rev Genet. 2016;17(2):71–80. doi: 10.1038/nrg.2015.8 26688196

[79] Edgar RC. Muscle5: High-accuracy alignment ensembles enable unbiased assessments of sequence homology and phylogeny. Nat Commun. 2022;13(1):6968. doi: 10.1038/s41467-022-34630-w 36379955 PMC9664440

[80] Stuart T, Butler A, Hoffman P, Hafemeister C, Papalexi E, Mauck WM 3rd, et al. Comprehensive Integration of Single-Cell Data. Cell. 2019;177(7):1888-1902.e21. doi: 10.1016/j.cell.2019.05.031 31178118 PMC6687398

[81] Trapnell C, Cacchiarelli D, Grimsby J, Pokharel P, Li S, Morse M, et al. The dynamics and regulators of cell fate decisions are revealed by pseudotemporal ordering of single cells. Nat Biotechnol. 2014;32(4):381–6. doi: 10.1038/nbt.2859 24658644 PMC4122333

[82] Qiu X, Hill A, Packer J, Lin D, Ma Y-A, Trapnell C. Single-cell mRNA quantification and differential analysis with Census. Nat Methods. 2017;14(3):309–15. doi: 10.1038/nmeth.4150 28114287 PMC5330805

[83] Satpathy AT, Granja JM, Yost KE, Qi Y, Meschi F, McDermott GP, et al. Massively parallel single-cell chromatin landscapes of human immune cell development and intratumoral T cell exhaustion. Nat Biotechnol. 2019;37(8):925–36. doi: 10.1038/s41587-019-0206-z 31375813 PMC7299161

[84] Wang J, Chai Y, Yang J, Ye Y, Luo J, Yin H, et al. Dissecting the role of transcription factor AP2-M in Babesia asexual replication. FASEB J. 2024;38(20):e70119. doi: 10.1096/fj.202400127RRR 39441647

