# Supplementary figures and images for: Comparative genomics, integrated with single-cell sequencing and genetic analyses, reveal roles of transcription factor AP2-M2 in asexual replication of Babesia parasite
Source: PLoS Pathog. 2025 Nov 13;21(11):e1013699. doi: 10.1371/journal.ppat.1013699 (PMC12626333; doi:10.1371/journal.ppat.1013699)

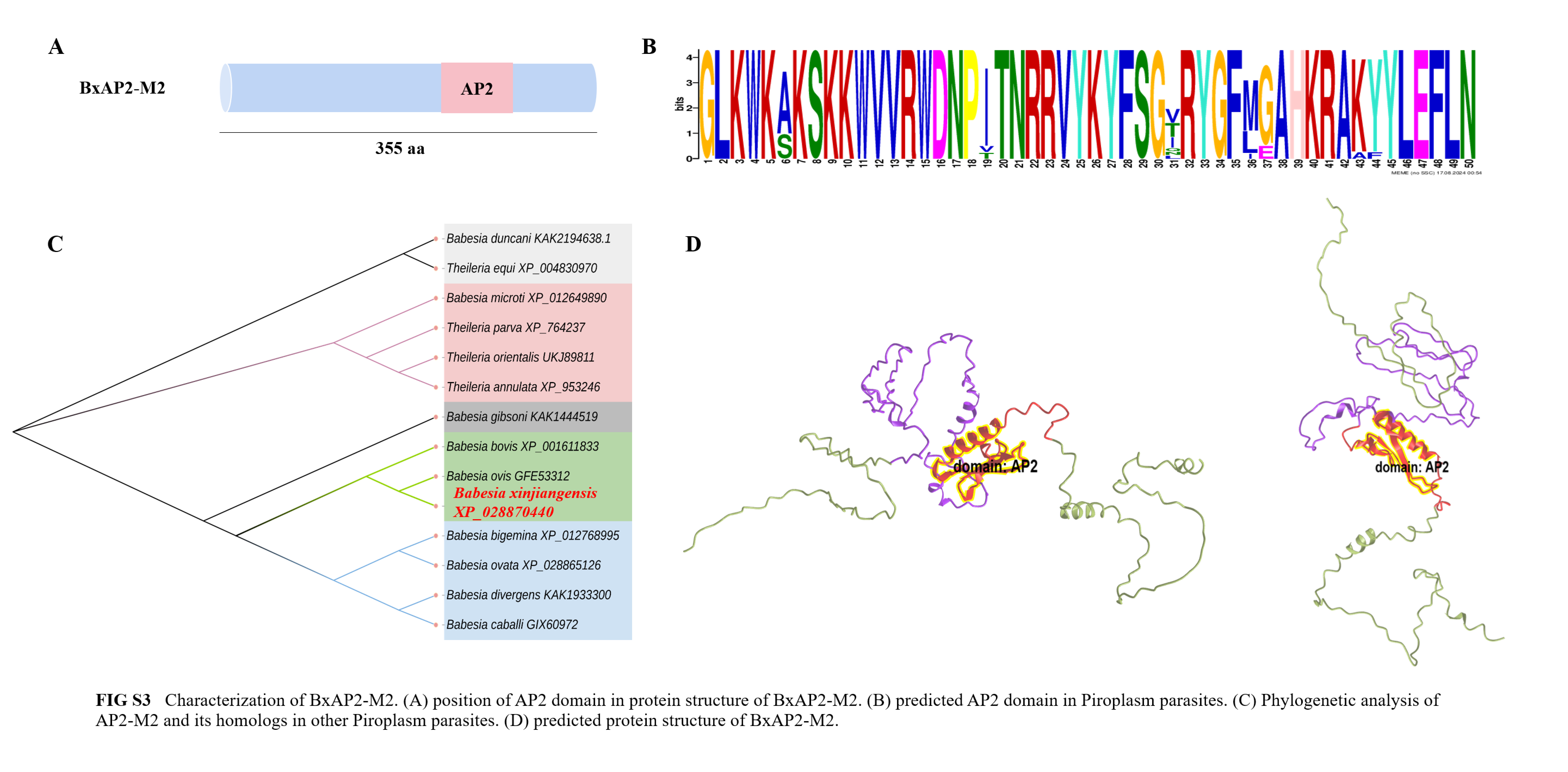

Supplement: S3 Fig — (A) Position of AP2 domains in protein structure of BxAP2-M2. (B) Predicted AP2 domain in Piroplasm parasites. (C) Phylogenetic analysis of BxAP2-M2 and its homologues in other piroplasm parasites. (D) Predicted protein structure of BxAP2-M2. (TIF) [file ppat.1013699.s003.tif]

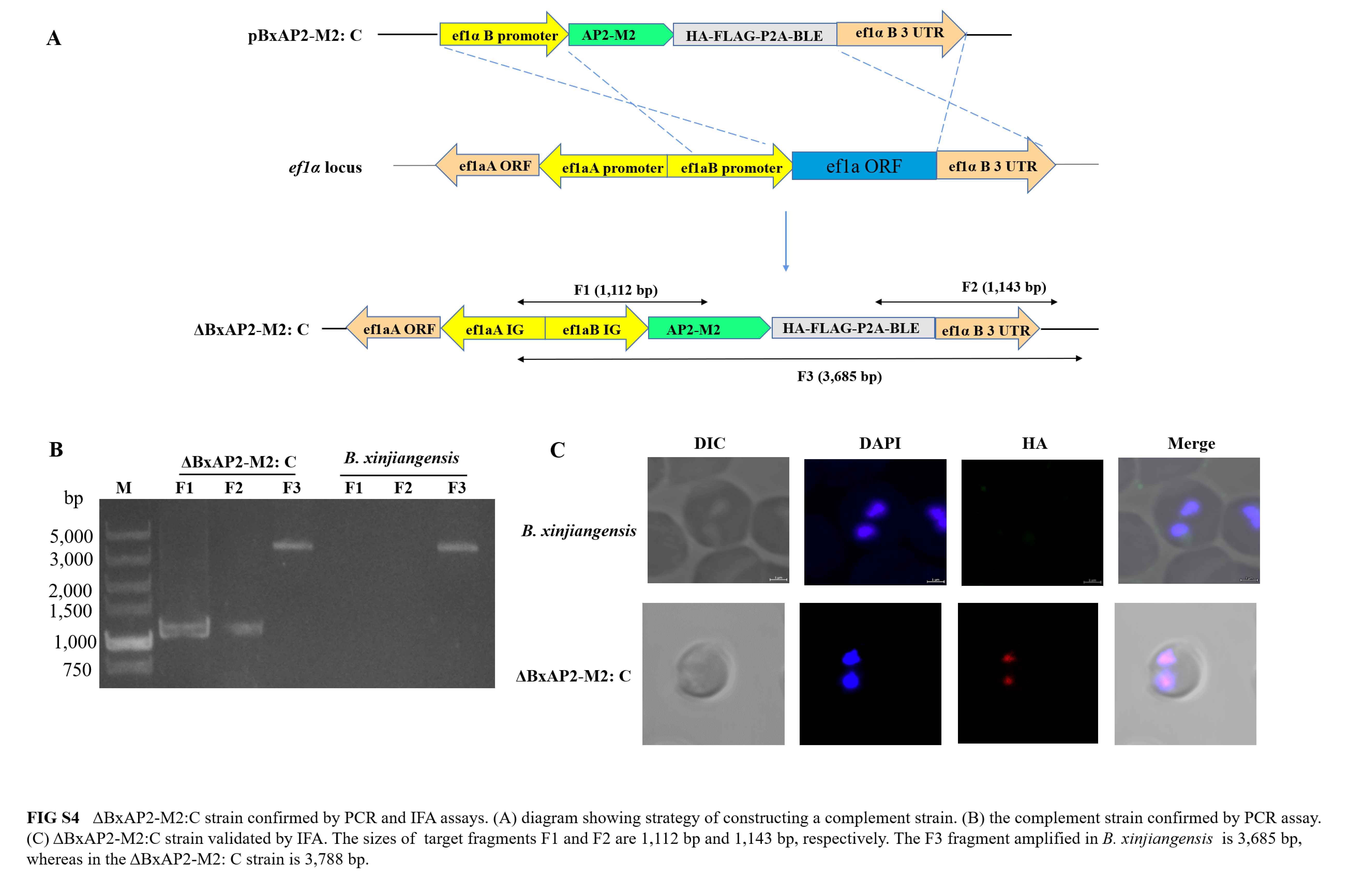

Supplement: S4 Fig — (A) Diagram showing strategy of constructing a complement strain. (B) Complemented strain confirmed with PCR assay. (C) ΔBxAP2-M2:C strain validated with immunofluorescence assay. (TIF) [file ppat.1013699.s004.tif]
